# Impact of the Recognition Part of Dipeptidyl Nitroalkene Compounds on the Inhibition Mechanism of Cysteine Proteases Cruzain and Cathepsin L

**DOI:** 10.1021/acscatal.3c01035

**Published:** 2023-04-24

**Authors:** Kemel Arafet, Santiago Royo, Tanja Schirmeister, Fabian Barthels, Florenci V. González, Vicent Moliner

**Affiliations:** † Dipartimento di Scienze degli Alimenti e del Farmaco, Università degli Studi di Parma, 43124 Parma, Italy; ‡ BioComp Group, Institute of Advanced Materials (INAM), Universitat Jaume I, 12071 Castelló, Spain; § Departament de Química Inorgànica i Orgànica, Universitat Jaume I, 12071 Castelló, Spain; ∥ Institute of Pharmaceutical and Biomedical Sciences, Johannes Gutenberg-Universität, 55128 Mainz, Germany

**Keywords:** cysteine proteases, cathepsin L, cruzain, dipeptidyl nitroalkenes, inhibitory activities, QM/MM, free energy surfaces, in vitro activities

## Abstract

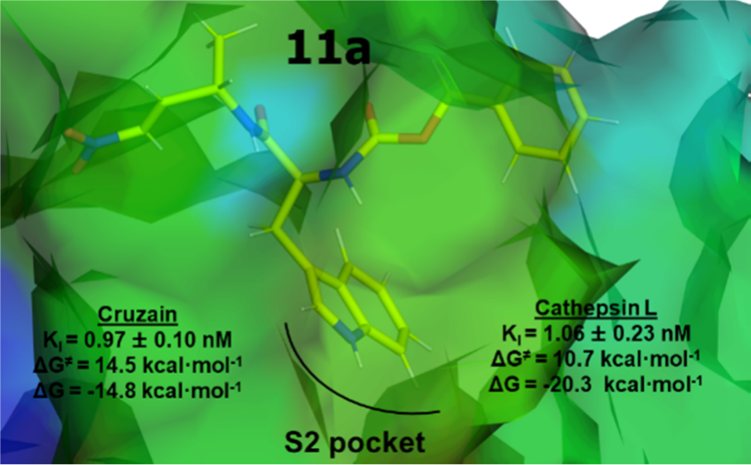

Cysteine proteases (CPs) are an important class of enzymes, many of which are responsible for several human diseases. For instance, cruzain of protozoan parasite *Trypanosoma cruzi* is responsible for the Chagas disease, while the role of human cathepsin L is associated with some cancers or is a potential target for the treatment of COVID-19. However, despite paramount work carried out during the past years, the compounds that have been proposed so far show limited inhibitory action against these enzymes. We present a study of proposed covalent inhibitors of these two CPs, cruzain and cathepsin L, based on the design, synthesis, kinetic measurements, and QM/MM computational simulations on dipeptidyl nitroalkene compounds. The experimentally determined inhibition data, together with the analysis and the predicted inhibition constants derived from the free energy landscape of the full inhibition process, allowed describing the impact of the recognition part of these compounds and, in particular, the modifications on the P2 site. The designed compounds and, in particular, the one with a bulky group (Trp) at the P2 site show promising *in vitro* inhibition activities against cruzain and cathepsin L for use as a starting lead compound in the development of drugs with medical applications for the treatment of human diseases and future designs.

## Introduction

Cysteine proteases (CPs) are an important class of enzymes responsible of several human diseases.^1−5^ For instance, cruzain CP is essential for the metabolism of the protozoan parasite *Trypanosoma cruzi*,^2,6^ responsible for the Chagas disease.^7^ Parasitic rhodesain CP is expressed by protozoa *Trypanosoma bruceirhodesiense*, which is responsible for the African sleeping sickness.^3^ The role of cathepsin L is well-known in several human diseases, including liver fibrosis,^8^ insulitisinsulitis,^9^ cancers,^10−12^ or the recent identification of cathepsin L as a potential target for the treatment of COVID-19 due to its critical role in SARS-CoV-2 entry into the host cells.^13−16^ Therefore, CPs have become attractive targets for the development of new inhibitors due to their medicinal properties.

Several lead compounds have been reported to show good inhibition activity against CPs.^17−27^ We proposed dipeptidyl nitroalkenes^17^ and enoates^18^ as potent Michael acceptor (MA) inhibitors of cruzain and rhodesain CPs. Both MA inhibitors are based on drug candidate *N-*methylpiperazine–Phe–homoPhe–vinylsulfone–phenyl, called K11777, a dipeptidyl vinylsulfone that inactivates CP in an irreversible manner.^28^ K11777 has demonstrated efficacy in preclinical trials in both mice and dogs;^29^ however, its preclinical evaluation has stalled for various contraindications.^30,31^ Gerwick and co-workers designed analogues of gallinamide A, an MA inhibitor originally isolated with a modest antimalarial activity, as potent inhibitors of cruzain and cathepsin L CPs.^19^ Meek and co-workers developed a novel class of reversible MA inhibitors of cruzain, peptidomimetic vinyl heterocyclic inhibitors. Despite their reversible character, these inhibitors are potent time-dependent inhibitors of cruzain.^20^ Ferreira and co-workers proposed several quinazolines as potent cruzain and rhodesain inhibitors.^24^ Recently, the dual inhibition of a peptide aldehyde called MG-132 against SARS-CoV-2 main protease (M^pro^/3CL^pro^) and human cathepsin L has been proposed by Storici and co-wokers.^27^

**Scheme 1:**
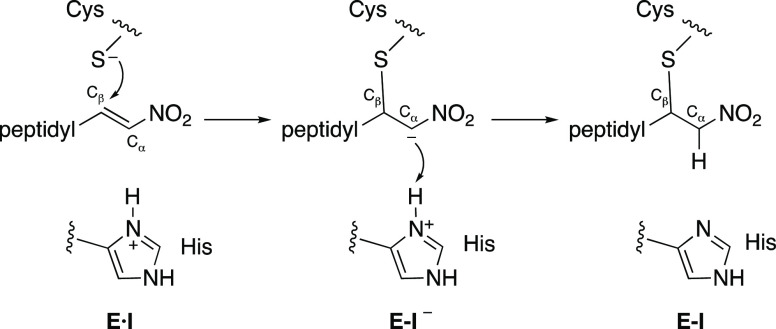
Proposed General Inhibition Mechanism of Cysteine Proteases by Dipeptidyl Nitroalkenes

With regard to the inhibition mechanism of cruzain and cathepsin L CPs, only a few studies have reported using computational tools including the effects of the protein environment.^32−38^ Montanari and co-workers have studied the inhibition mechanism of cruzain by several dipeptidyl nitrile inhibitors.^34,36^ In all cases, the calculated free energy profiles show that the Cys25 nucleophilic attack and His159 proton transfer take place in a concerted manner. Likewise, in our laboratory, we have employed quantum mechanics/molecular mechanics (QM/MM) simulations to study the inhibition mechanism of CPs by different families of peptidic inhibitors.^32,33,35,37,39−41^ In the case of nitroalkene inhibitors, the inhibition mechanism proceeds by a Michael addition mechanism, the anionic CysS^–^ residue first attacks the β-carbon of the MA inhibitor, and later the proton from the active site HisH^+^ residue is transferred to the α-carbon of the MA inhibitor to form a thioether derivative (see Scheme 1). The stepwise character of the inhibition mechanisms of CPs by nitroalkene inhibitors has been proposed using different enzymes and MA inhibitors,^35,40^ in contrast to, for instance, the concerted inhibition mechanisms of CP proposed by Lameira and co-workers when using alkyne- and nitrile-based inhibitors.^34,36,42^ The importance of the QM/MM simulations to investigate the reactivity of covalent inhibitors within their protein targets has been demonstrated, particularly for the design of inhibitors bearing Michael acceptors.^32,33,35,37,39−41,43−46^

**Figure 1:**
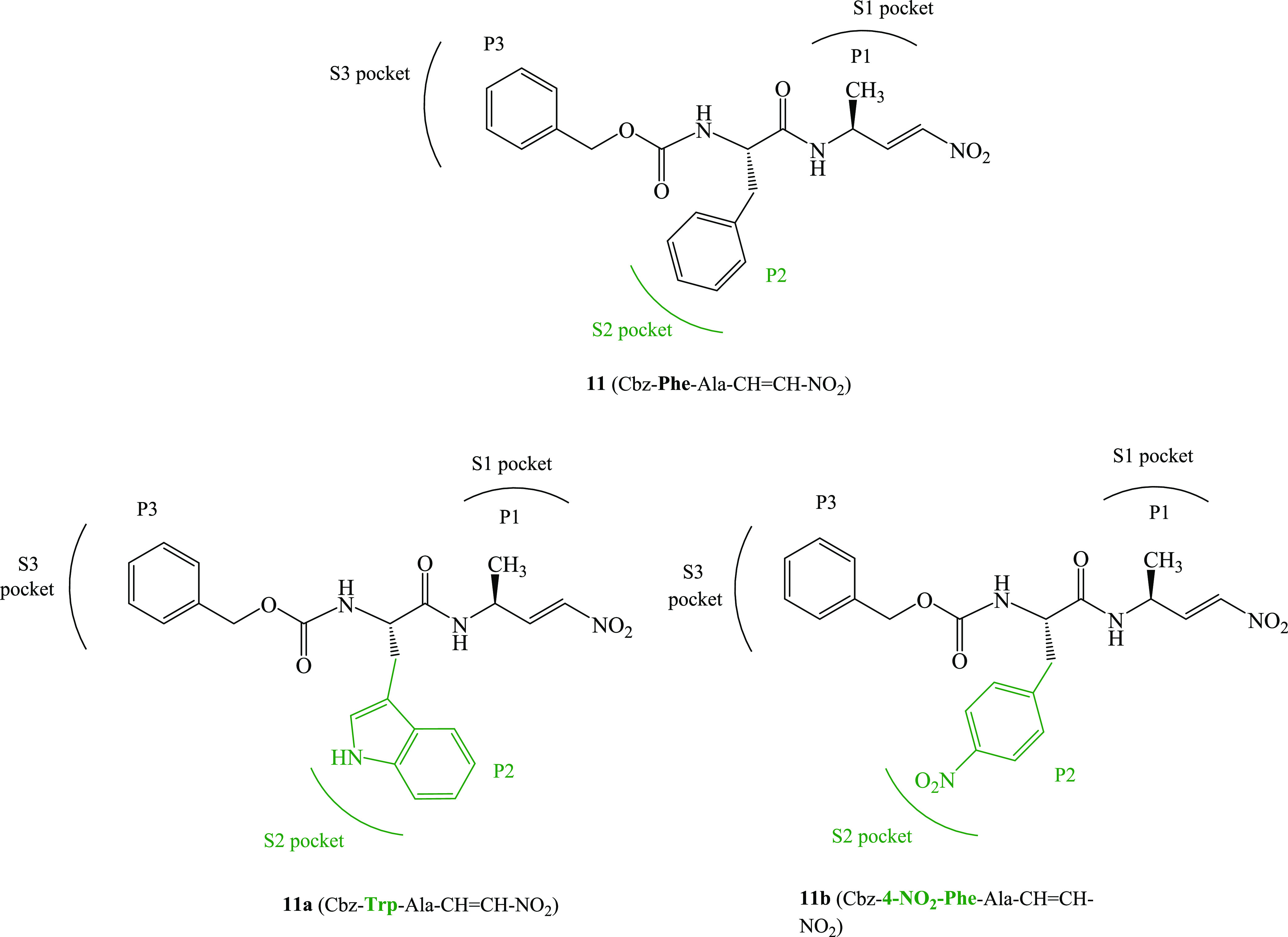
Chemical structures of known (**11**), from ref (17), and proposed (**11a** and **11b**) dipeptidyl nitroalkenes inhibitors of cruzain and cathepsin L cysteine proteases.

For many years, it has been demonstrated that the inhibition of CPs depends on the recognition part of the inhibitor that directs the inhibitor to the target enzyme displaying a peptidic framework to resemble the structural and functional motives of the natural substrate.^17−20,22,23,37,47−55^ Gerwick and co-workers concluded that the activity to cathepsin L increases when the P1′ position is a large aliphatic or aromatic residue, and the highest affinity binding is found with the presence of both Phe at the P1 and Leu at the P1′ position (according to commonly employed Berger and Schechter nomenclature, amino acid residues P1··· Pn and P1′··· Pn′ of the inhibitor are complementary to S1··· Sn and S1′··· Sn′ sites of the active site, respectively, being the scissile bond the one between P1 and P1′) (Figure 1).^19^ Larsen and co-workers found that variations in the P3 position of the triazine nitrile inhibitors had little impact on their potency or selectivity, with most of the potency and selectivity caused by efficient binding at the S2 pocket of human cathepsin L.^23^ In the case of cruzain, the S2 pocket is able to bind both basic and hydrophobic residues, especially due to the presence of the Glu205 residue in this pocket.^47^ Glu205 can adopt either inhibitor-directed or solvent-directed conformation, depending on the chemical character of the P2 side chain.^47^ Zhai and Meek investigated the role of Glu205 in recognition of the P2 position.^52^ Even though wild-type cruzain recognizes better the Cbz–Phe–Arg–AMC substrate than the E205A mutant, the values of k_cat_ are very similar. However, mutation of Glu205 considerably affects the kinetic parameters of substrates bearing an Arg residue at the P2 position.^52^

We proposed the dipeptidyl nitroalkene family of MA as potent reversible covalent inhibitors of cruzain and rhodesain CPs, being the Cbz–Phe–Ala–CH=CH–NO_2_ inhibitor the most potent one (compound **11**, as shown in Figure 1).^17^ In our laboratory, we studied the inhibition mechanism of cruzain and cathepsin L CPs by compound **11** applying QM/MM simulations.^35^ The analysis of the interactions between the S2 pocket of these enzymes and the Phe residue (P2 position) of the inhibitor reveals the important role of Glu205 in the inhibition mechanism of cruzain. Moreover, the role of Leu67 and Met68 appears to be important in the inhibition of cruzain and Glu159 plays an important role in the inhibition of human cathepsin L. As noted above, in papain-family cysteine proteases, the P2 position can be a key determinant of specificity.

**Scheme 2:**
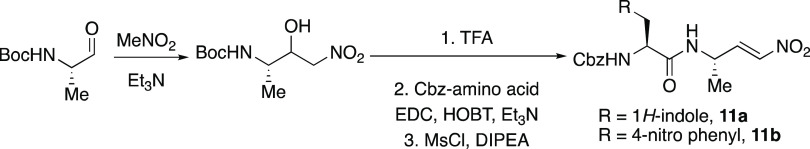
Preparation of Dipeptidyl Nitroalkenes

Herein, we report a combined experimental and computational study of the inhibition mechanisms of cruzain and cathepsin L CPs by dipeptidyl nitroalkenes **11a** and **11b** (see Figure 1). The aim of the study is to explore the influence of the affinity and selectivity of two different groups at the P2 site: a bulky group and a polar group. Based on our previous results with compound **11**,^35^ we have designed and tested two nitroalkene inhibitors to inhibit cruzain and cathepsin L CPs: compound **11a** with a bulky group at the P2 site and compound **11b** with a polar group (see Scheme 2). Thus, Phe at P2 was replaced by Trp (**11a**) and 4-NO_2_–Phe (**11b**). The analysis of the experimentally determined inhibition data together with the predicted kinetic data derived from free full energy landscape of the full inhibition process, computed in terms of the potential of mean force (PMF), and the interactions between the inhibitor and the protein allowed describing the impact of P2 modification on the inhibition mechanism of these CPs by dipeptidyl nitroalkenes, establishing the bedrock for future designs of more potent inhibitors of these two enzymes that are involved in human diseases. At this point, it is important to point out that although there is a concern about the toxicity of nitro groups, numerous examples of prodrugs and drugs containing nitro groups are commonly used in medicine.^56^ Anyway, for the future development of potential drugs based on these inhibitors, toxicity tests will be required.

## Methods

### Computational Methods

The cruzain–inhibitor molecular models were constructed from the X-ray crystal structure of cruzain from *T. cruzi* bound to Cbz–Tyr–Ala–CH_2_F with PDB code 1AIM,^47^ while the initial coordinates for building the cathepsin L–inhibitor models were taken from the X-ray crystal structure of human cathepsin L bound to (2S,4*R*)-4-(2-chlorophenyl)sulfonyl-1-[1-(5-chlorothiophen-2-yl)cyclopropyl] carbonyl-*N*-[1-(iminomethyl)cyclopropyl]pyrrolidine-2-carboxamide with PDB code 2XU3;^57^ in both cases, the respective inhibitors were replaced by nitroalkene inhibitors **11a** and **11b**. The missing hydrogen atoms of the X-ray structures were added at pH 7 using the tLEaP module of the Amber Tools program^58^ within the pKa values of the titratable residues previously calculated within the empirical PROPKA 3.1 program.^59^ A total of seven and eight Na^+^ counterions were added for cruzain and cathepsin L models, respectively. Finally, the systems were solvated in orthorhombic boxes of TIP3P^60^ water molecules with the following sizes: cruzain 69.5 Å × 71.5 Å × 79.8 Å and cathepsin L 69.8 Å × 80.7 Å × 80.4 Å. Initial energy minimizations were carried out, followed by series of molecular dynamics (MD) in the NVT ensemble with the AMBER ff03 force field.^61^

The reaction was studied using a QM/MM approach from the equilibrated structures. The QM region was described with the AM1d semiempirical Hamiltonian^62^ and M06-2X functional.^63^ M06-2X is a hybrid functional developed and recommended by Truhlar and co-workers for the study of main group thermochemistry, kinetics, and noncovalent interactions, and, according to our previous tests and experience that include the study of CP proteolysis reactions and inhibition,^32,33,35,37,39−41,64,65^ it is a good choice when combined with the 6-31+G(d,p) basis set.^66^ Moreover, Rowley and co-workers demonstrated that the M06-2X functional describes reasonably well the reaction between a model thiolate and Michael acceptor inhibitors.^67^ The optimized OPLS-AA^68^ and TIP3P^60^ classical force fields were used to treat the protein and solvent water molecules, respectively, as implemented in the fDynamo library.^69^ After potential energy surfaces (PESs) were computed, the appropriate distinguished reaction coordinates were explored, and free energy surfaces (FESs) for each of the chemical steps were calculated in terms of potentials of mean forces (PMFs) at the AM1d/MM level and subsequently improved at the M06-2X/MM level by means of spline corrections. Structures selected from the quadratic regions of the corrected FESs were used as starting point to optimize the transition state at the M06-2X/6-31+G(d,p)/MM level to confirm the quality of the employed strategy and the robustness of the results. A detailed description of the computational methods can be found in the Supporting Information.

## Experimental Methods

### Experimental Procedure for the Preparation of Dipeptidyl Nitroalkenes

For the synthesis of the inhibitors, a straightforward route was applied based upon previous results.^17^

First, a nitroaldol reaction between *N*-*tert*-butoxycarbonyl alaninal and nitromethane afforded a mixture of nitroaldols. The resulting compounds were submitted to a three-step sequence of deprotection, coupling with corresponding *N*-benzylocarbonyl amino acid, and then elimination (Scheme 2). In particular, nitromethane (6 mmol) and triethyl amine (42 mL, 0.3 mmol) were added to an ice-bath cold solution of *tert*-butoxycarbonyl amino aldehyde (1 mmol) in dichloromethane (1 mL). The resulting mixture was stirred at 23 °C for 8 h, then quenched with saturated ammonium chloride aqueous solution (25 mL), and extracted with dichloromethane (3 × 15 mL); the organic layers were washed with 1 M HCl solution (15 mL), saturated sodium hydrogen carbonate aqueous solution (15 mL), and brine (15 mL); dried (Na_2_SO_4_); and concentrated. The crude oil was directly submitted to the next step without any further purification. The resulting mixture of nitroaldols was dissolved in dichloromethane (1 mL), cooled with an ice bath, and then trifluoroacetic acid (1.5 mL, 20 mmol) was added. The resulting mixture was stirred at 23 °C for 30 min and then directly concentrated under vacuum. The resulting crude was dissolved in dichloromethane (10 mL). Then, benzyloxycarbonyl amino acid (1.1 mmol), hydroxybenzotriazole (168 mg, 1.1 mmol), triethyl amine (558 mL, 4 mmol), and EDC (211 mg, 1.1 mmol) were sequentially added. The resulting mixture was stirred at 23 °C for 8 h, then quenched with saturated ammonium chloride aqueous solution (25 mL), and extracted with dichloromethane (3 × 15 mL); the organic layers were washed with 1 M HCl solution (15 mL), saturated sodium hydrogen carbonate aqueous solution (15 mL), and brine (15 mL); dried (Na_2_SO_4_); and concentrated. The crude oil was directly submitted to the next step without any further purification. The resulting diastereomeric mixture of dipeptidyl nitroaldols was dissolved in dichloromethane (10 mL). The resulting mixture was treated with diisopropyl ethyl amine (697 mL, 4 mmol) and methanesulfonyl chloride (155 mL, 2 mmol) and then stirred at 23 °C for 2 h. Then, it was quenched with saturated ammonium chloride aqueous solution (25 mL) and extracted with dichloromethane (3 × 15 mL); the organic layers were washed with 1 M HCl solution (15 mL), saturated sodium hydrogen carbonate aqueous solution (15 mL), and brine (15 mL); dried (Na_2_SO_4_); and concentrated. The crude oil was purified through silica gel chromatography (hexane/ethyl acetate, 9/1 and 7/3) to afford the desired dipeptidyl nitroalkene.

Dipeptidyl nitroalkenes **11a** and **11b** were prepared as described above and fully characterized (see the Supporting Information for spectral and characterization data).

### Enzyme Assays

The inhibitory activity of compounds **11a** and **11b** was tested against recombinant cruzain and cathepsin L enzymes as reported previously^17,70^ In particular, recombinantly expressed cruzain (0.9 mg/mL) was diluted 1:600 in enzyme buffer (50 mM sodium acetate, pH 5.5, 5 mM EDTA, 200 mM NaCl, and 2 mM DTT) and preincubated for 1 h at room temperature. Enzymatic reactions were carried out with either 5 μL of cruzain stock solution or 5 μL of human cathepsin L (Calbiochem, 1:100 dilution in enzyme buffer) in 180 μL of assay buffer (50 mM sodium acetate, pH 5.5, 5 mM EDTA, 200 mM NaCl, 0.005% Brij). Ten microliters of the inhibitors (final conc.: 1000–0.78 nM) was added from DMSO stock solutions. Reactions were initiated by the addition of 5 μL of Cbz–Phe–Arg–AMC in DMSO (cruzain, 5 μM; cathepsin L, 6.25 μM). Enzymatic reactions were monitored for 30 min with a Tecan Spark microplate reader (λ_ex_, 380 nm/λ_em_, 460 nm). The measurements were performed in triplicate.

## Results and Discussion

**Scheme 3:**
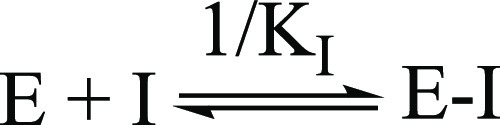
Inhibition Equilibrium

The desired inhibitors, **11a** and **11b**, were obtained with high yield and purity (see the Supporting Information for details) and submitted to in vitro testing with recombinant cysteine proteases cruzain and cathepsin L (see Table 1). The determination of the inhibition constant (*K*_I_) revealed that these compounds are sub-nanomolar potent inhibitors of these enzymes. *K*_I_ is the dissociation constant describing the binding affinity between the inhibitor and the enzyme, as shown in Scheme 3.

**Figure 2:**
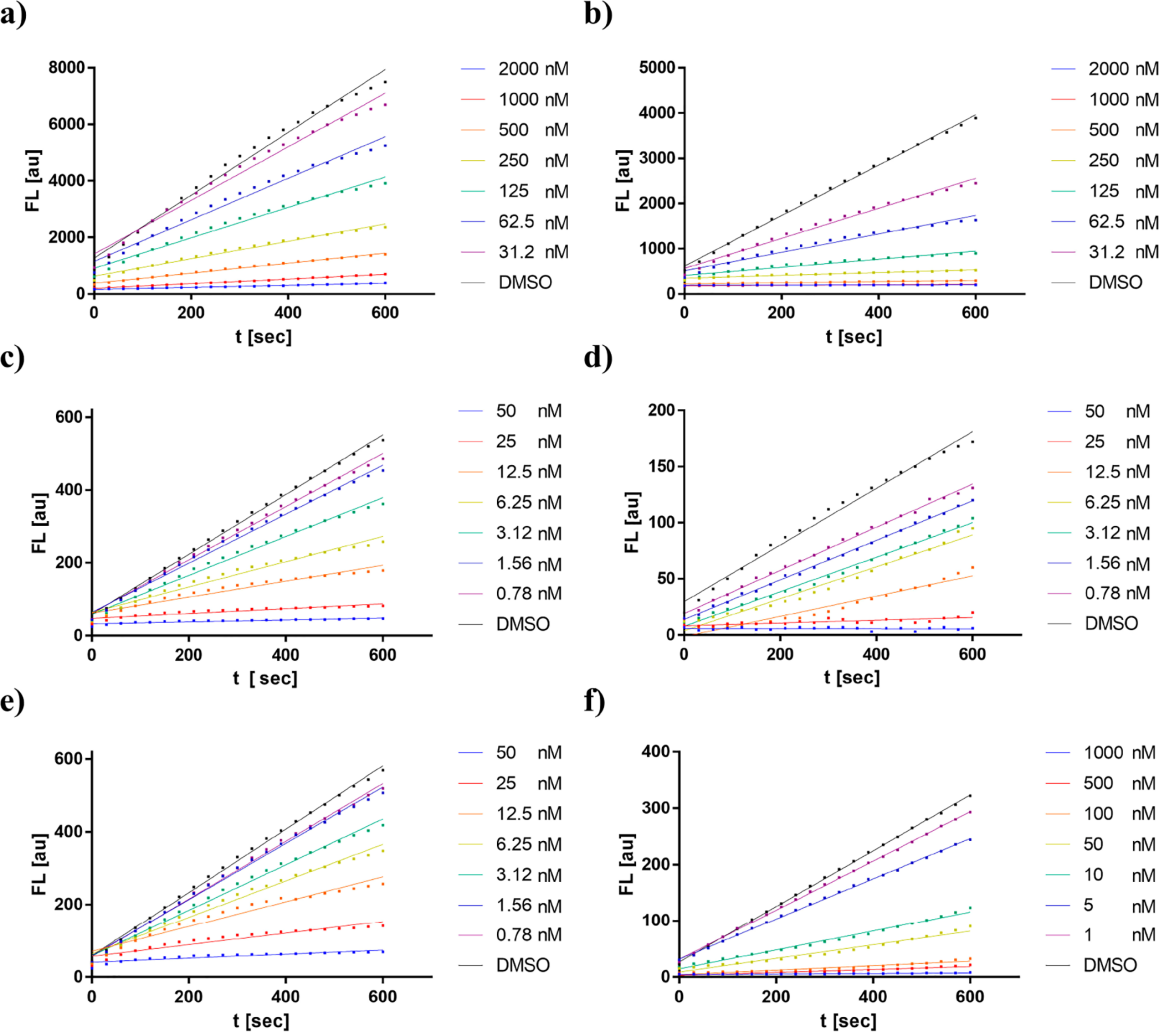
Fluorometric enzyme assay with varying concentrations of compound **11** with (a) cruzain (*K*_I_ = 0.44 nM) and (b) human cathepsin L (*K*_I_ = 11.0 nM), compound **11a** with (c) cruzain (*K*_I_ = 0.97 nM) and (d) human cathepsin L (*K*_I_ = 1.06 nM), and compound **11b** with (e) cruzain (*K*_I_ = 2.28 nM) and (f) human cathepsin L (*K*_I_ = 3.88 nM).

The straight lines of the fluorometric enzyme assays on cruzain and cathepsin L with varying concentrations of compounds **11a** and **11b** confirm the reversible mode of inhibition (Figure 2). Compound **11a** with l-alanine in the P1 position and l-tryptophane in P2 proved to be the most potent inhibitor (cruzain: *K*_I_ = 0.97 nM). According to the values reported in Table 1, both **11a** and **11b** should be slightly less potent inhibitors of the human off-target cathepsin L than cruzain. However, considering the minimal differences and the uncertainty of the reported values, despite the conserved trend, no significant specificity for cruzain or cathepsin L must be expected between compounds **11a** and **11b**.

**Table 1:** Inhibition Data, Expressed as Dissociation Constant *K*_I_ (nM) for Compounds **11**, **11a**, and **11b** Tested with Recombinant Cysteine Proteases Cruzain and Cathepsin L

compound	Cruzain	Cathepsin L
**11**^a^	0.440 ± 0.023	11.00 ± 3.10
**11a**	0.97 ± 0.10	1.06 ± 0.23
**11b**	2.28 ± 0.51	3.88 ± 0.30

a Data from ref (17).

Keeping in mind future possible developments of drugs based on these compounds, as commented in the Introduction section, toxicities and physicochemical properties should be measured. In this regard, the logP values (calculated by ChemBioDraw Ultra 13 software) for inhibitors **11**, **11a**, and **11b** are 1.89, 1.61, and 1.85, respectively. These values make them reasonable candidates for drug development, according to Lipinski’s rule of 5 recommendations.^71^ On the other side, keeping in mind that these compounds are peptide-like and consequently susceptible to proteolytic degradation, and despite peptide drugs have been approved worldwide since 2000 and many more are in clinical phases,^72^ one can envision some modifications of the structure like replacement of L-amino acids by D-amino acids or more hydrolytically stable peptide isosters for future development as drugs.

**Figure 3:**
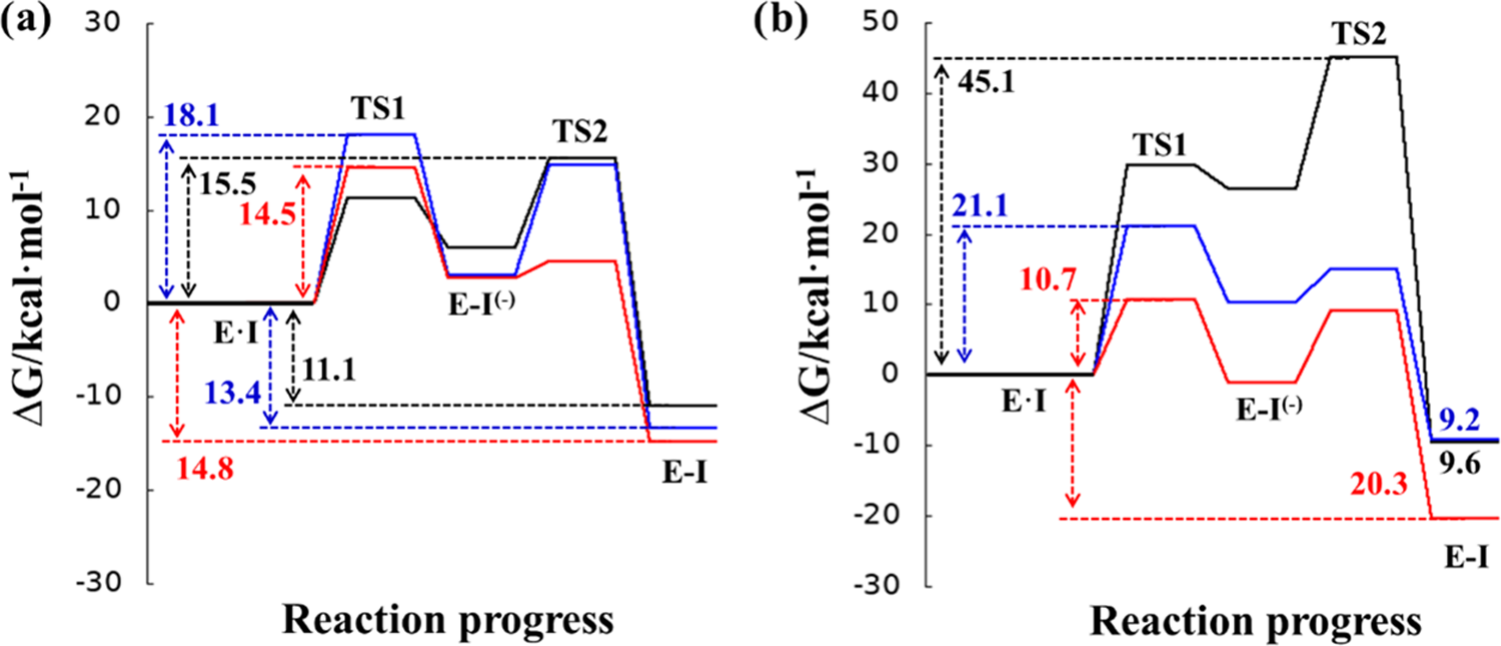
M06-2X/6-31+G(d,p):AM1d/MM free energy profiles obtained with umbrella sampling MD simulations for the inhibition mechanisms of the cysteine protease cruzain (a) and cathepsin L (b) by dipeptidyl nitroalkenes **11** (black line),^35^**11a** (red line), and **11b** (blue line). The FESs generated to provide these energy profiles are given in the Supporting Information (see Figures S4–S7), where the selected reaction coordinates are specified.

The computational study of the inhibition of cruzain and cathepsin L CPs by compounds **11a** and **11b** was initiated by the generation of the FESs in the proposed inhibition mechanism (see Scheme 1), prior to carrying out a deep analysis of the inhibition process with the two proposed compounds. In particular, the interatomic distances between SG of Cys25 and the C_β_ atom of the inhibitors and the antisymmetric combination of distances defining proton transfer from the N3 atom of His (His159 and His163 for cruzain and cathepsin L, respectively) to C_α_ were used as reaction coordinates (see the Methods section and the Supporting Information for details). The corresponding FESs computed in terms of PMFs at the M06-2X/6-31+G(d,p):AM1d /MM level are given in the Supporting Information Figures S4–S7. The resulting free energy profiles are shown in Figure 3, including those corresponding to dipeptidyl nitroalkene **11** recently obtained in our laboratory.^35^ As observed in Figure 3, the stepwise character of the inhibition mechanism proposed in Scheme 1 is confirmed in all cases:^35,40^ first, Cys25 of the protein attacks the C_β_ atom of the dipeptidyl nitroalkene, leading to a stable intermediate **E-I**^**(−)**^, and then the proton from His159 is transferred to the C_α_ atom of the inhibitor, forming the **E-I** covalent adduct.

Our previous results indicate that **11** should be an efficient inhibitor of cruzain with a slightly lower, but measurable, inhibitory activity for human cathepsin L,^35^ in total agreement with the experimental results previously reported by us.^17^ Thus, a comparative analysis between the reactivity of the three dipeptidyl nitroalkenes toward cruzain and cathepsin L CPs can be based on the obtained free energy profiles shown in Figure 3. Thus, the substitution of Phe at the P2 position by Trp and the 4-NO_2_–Phe fragment does not result in a considerable improvement in the efficiency of the inhibition process of cruzain (see Figure 3a). The activation free energy decreases by only 1.0 kcal·mol^–1^ for **11a** and increases by 2.6 kcal·mol^–1^ for **11b**. Although all cruzain inhibition processes are exergonic, the stability of the final **E-I** covalent complexes of the inhibition mechanisms decreases slightly in the following order: **11a** > **11b** > **11**. Considering the experimental values of *K*_I_ reported in Table 1, the computationally predicted trend on the reaction free energies between **11a** and **11b** agrees with the experimental data. However, in the case of compound **11**, it appears that other contributions apart from the pure thermodynamic equilibrium between reactant complex **E·I** and covalent complex **E-I** must be responsible for the final *K*_I_ values. The free energy barriers of **11** and **11a** are almost indistinguishable (14.5 and 15.5 kcal·mol^–1^), considering the error associated with the employed methodology that, just from the statistical uncertainty from MD sampling, is assumed to be around 1 kcal·mol^–1^.^73−75^ These values are, however, smaller than the value obtained for the reaction of inhibition with **11b** (18.1 kcal·mol^–1^). These kinetic results, together with the energy barriers corresponding to the inverse process, of the decomposition of **E-I** back to the **E·I** reactant complex (25.6, 29.3, and 31.5 kcal·mol^–1^ for **11**, **11a**, and **11b**, respectively) agree with the trend of *in vitro* determined *K*_I_ values, despite the reported differences being small. At this point, it is also important to point out that the free energy profiles shown in Figure 3 correspond to the chemical process from the **E·I** reactant complex to the final covalent complex, **E-I**, while the *K*_I_ values reported in Table 1 refer to the complete thermodynamic equilibrium of the inactivation process starting from the solvent-separated species, **E** + **I** in solution, as described in Scheme 3. Anyway, according to our computational results, it appears that the trends of the inhibition of cruzain and cathepsin L with the different compounds may be dictated by the chemical steps, despite no significant differences being experimentally observed, as discussed above.

Regarding the inhibition process of cathepsin L, the overall conclusion from Figure 3b is that both dipeptidyl nitroalkenes **11a** and **11b** show better kinetic and thermodynamic values for the inhibition process than compound **11**, which is in qualitative agreement with our experimental data (see Table 1). A significant reduction in the activation of free energy takes place when employing compounds **11a** and **11b**, by comparison with originally designed **11**. In particular, this is reduced by 34.4 kcal·mol^–1^ for **11a** and 24.0 kcal·mol^–1^ for **11b**; therefore, we predict that the new compounds should have a higher inhibitory potency than compound **11**, especially in the case of compound **11a**, which should be a potent inhibitor of cathepsin L cysteine protease, according to the computed kinetic parameters. Moreover, strong stabilization of the **E-I** covalent complex occurs during the inhibition of cathepsin L with this compound, compared to compound **11** (20.3 vs 9.6 kcal·mol^–1^). The difference of the activation free energy barrier between compound **11** (derived from our previous study carried out with the same computational methodology^35^) and compound **11a** or **11b** in cathepsin L is in qualitative agreement not only with the experimental decrease of the measured *K*_I_ values (despite by just one order of magnitude, as shown in Table 1) but also, as commented in the Introduction section, with previous studies showing the determinant effect on the specificity of the P2 position in inhibitors of papain-family cysteine proteases, including our own previous computational study.^23,47,52,76^ In this regard, the reactivity between a ligand and the active site of an enzyme very much depends not only on the warhead of the former but also on the complete pattern of interactions that are established between other parts of the inhibitor (the recognition part) and the protein. Changes in the recognition part can dramatically influence the binding of the compound. This can have a decisive effect on the pose with respect to the active site and, consequently, on exploring different reactive conformations. Changing a moiety of a ligand does not only influence the wave function (electronic distribution) of the ligand that can influence the inherent reactivity of the warhead but also can primally affect the protein–ligand relative orientation on the Michaelis complex, the transition states, and/or intermediates. Similar dramatic catalytic activity differences have been previously observed between compounds with very similar structures.^17,18,48^ In the present case, replacing the phenyl substituent in the P2 position of **11** with either a nitrophenyl (**11b**) or imidazolyl (**11a**) provokes different protein–ligand interaction patterns in the **E·I** reactant complex that can constraint the substrate in the active site in a nonfavourable reactive conformation (Figures S15 and S16 of the Supporting Information). In particular, these differences affect the interaction between the warhead and the P2 moieties in the S1′ and S2 pockets, respectively, and also between P3 and S3. On the other side, differences are also detected in **TS2** (Figure S17 of the Supporting Information). Thus, the distance between the proton of His163 and the acceptor Cα of the substrate is significantly longer in **11** (1.65 Å) than those in **11a** (1.50 Å) and **11b** (1.48 Å). In addition, the orientation of P2 in the S2 pockets in **TS2** of compound **11** is also different from those of **11a** and **11b** (Figure S17 of the Supporting Information). As discussed below, these geometrical differences agree with those detected in the quantitative analysis of the electrostatic and Lennard–Jones interactions, especially (but not only) those with Lys117 and Asp71.

Overall, the energetic data derived from our computational results show that compound **11a** should be a more efficient inhibitor of cathepsin L than compound **11**, in qualitative agreement with our experimental measurements of *K*_I_. Regarding compound **11b**, our calculations show stabilization of the covalent complex equivalent to that of **11** but significantly lower activation free energies. These results agree with the trend of the experimentally determined *K*_I_ values (Table 1): almost indistinguishable activities of the three inhibitors in cruzain (differences of reaction energies and activation energies of less than 4 kcal·mol^–1^) and slightly higher activity of compounds **11a** and **11b** than **11** in the inhibition of cathepsin L.

Analysis of the free energy profiles in Figure 3 can also be used to confirm the reversible vs irreversible character of the inhibitors. Traditionally, this classification is based on the values of the reaction energy (energy difference between **E-I** and **E·I**), and a value of ca. 22 kcal·mol^–1^ is considered the limit to distinguish them.^77^ However, as pointed out by Rowley and co-workers, the irreversible vs reversible character of the inhibitors, with potentially paramount importance for finding the optimal balance between efficacy and safety, also depends on the free energy barrier of the inverse process if the reaction is strongly exergonic.^78^ In this regard, recent studies on the reversibility for covalent cysteine protease inhibitors using QM/MM FES calculations predicted the reversible character of nitrile-based inhibitors based on reaction free energies of −11.8 kcal·mol^–1^ and an activation free energy of 17.3 kcal·mol^–1^.^42^ Following this criterion, all our tested compounds would behave as reversible covalent inhibitors. In our case, a covalent bond between Cys25 of the protein and the C_β_ atom of the dipeptidyl nitroalkene is formed in the intermediate **E-I**^**(−)**^, and consequently, the free energy barrier of the reverse process must be measured as the difference between **TS1** and **E-I**^**(−)**^. Thus, in agreement with the experimental evidence deduced from Figure 2, all three compounds would behave as reversible inhibitors in the two CPs.

**Figure 4:**
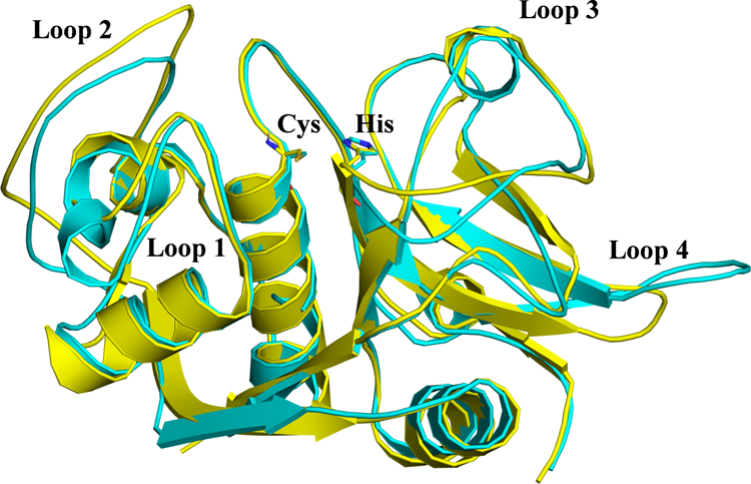
Overlay of the X-ray structures of cruzain (PDB code 1AIM, in yellow) and cathepsin L (PDB code 2XU3, in cyan). The cysteine and histidine active site residues are depicted in licorice.

**Figure 5:**
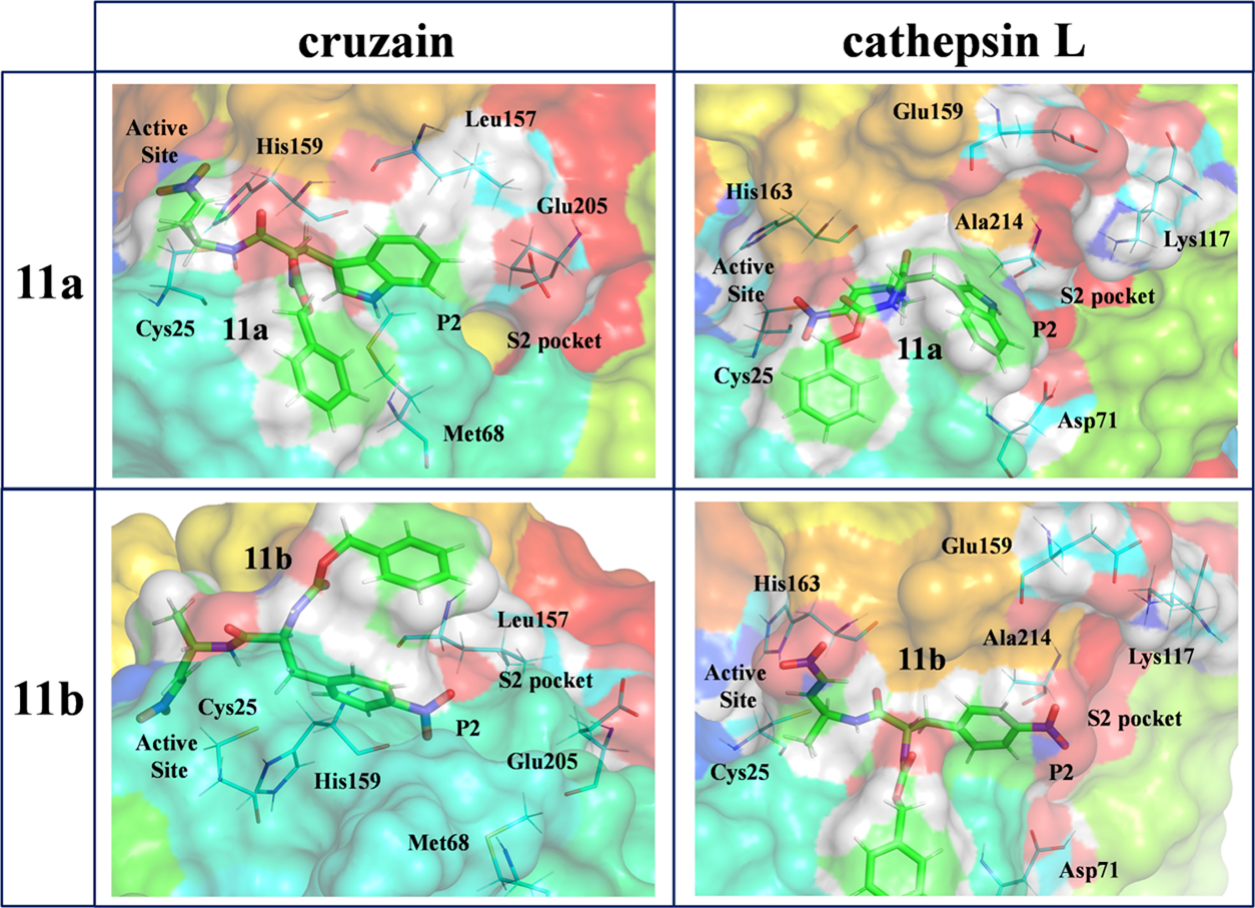
Representative structures of the active site of cruzain (left panels) and cathepsin L (right panels) in the **E·I** reactant complex of the inhibition processes with **11a** (top panels) and **11b** (bottom panels).

**Figure 6:**
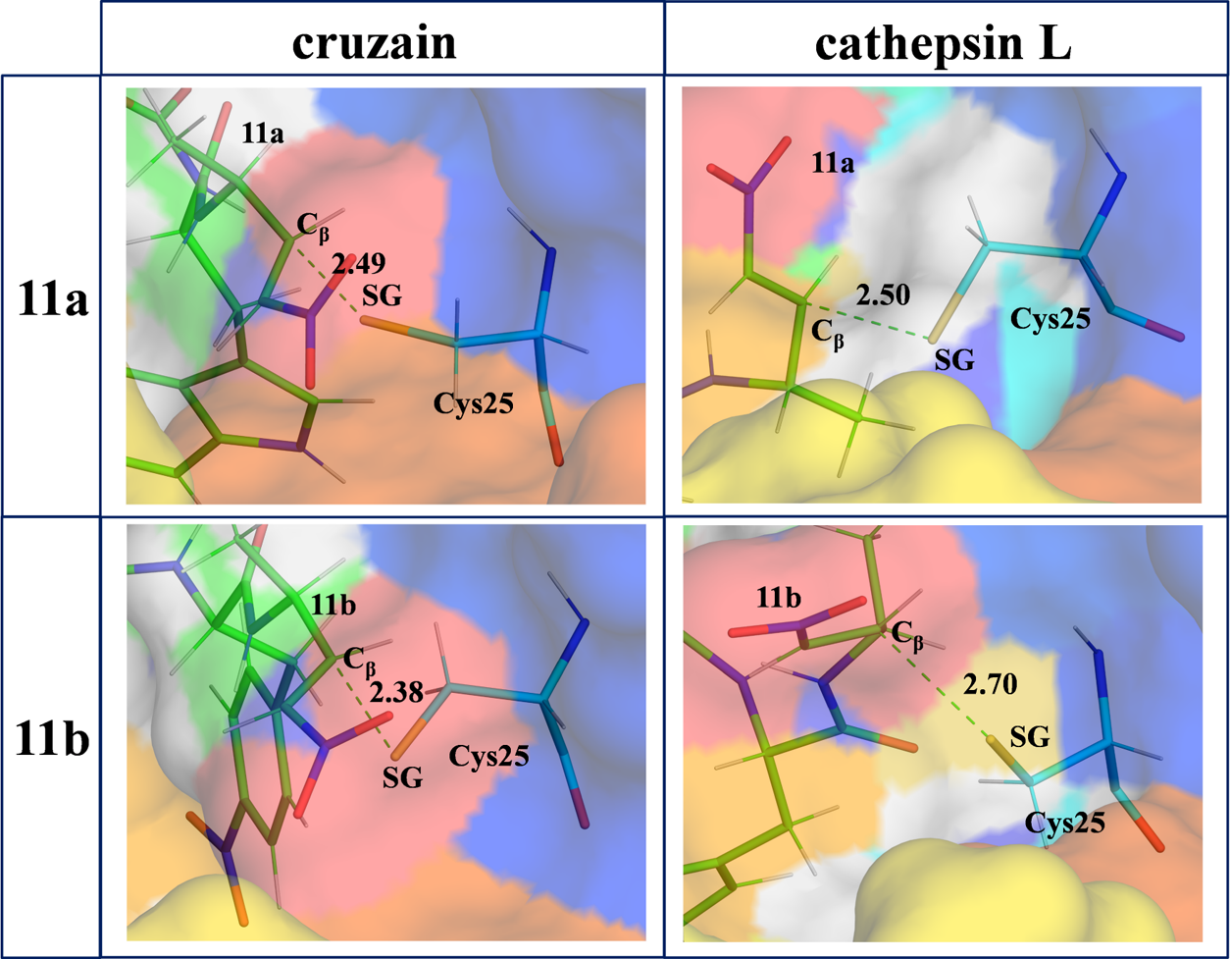
Details of the M06-2X/6-31+G(d,p)/MM optimized structures of TS1 located along the inhibition of cruzain (left panels) and cathepsin L (right panels) by **11a** (top panels) and **11b** (bottom panels). Key distances are in Å.

Thus, the next step of our study, prior to carrying out a deep analysis of inhibition, was to perform a comparative study of the structures of cruzain and cathepsin L. Figure 4 shows the overlay of the crystal structures of cruzain (PDB code 1AIM) and cathepsin L (PDB code 2XU3), while representative structures of the active sites in the **E·I** reactant complex state of the inhibition processes with **11a** and **11b** are shown in Figure 5. The corresponding representations of the **E-I** final states are shown in Figures S12 and S13 of the Supporting Information. Details of the active site of optimized transition-state structures at the M06-2X/6-31+G(d,p)/MM level are shown in Figures 6 and S14.

As shown in Figure 4, the structures of both enzymes are quite similar, including the relative position of the active site residues Cys and His. These structural similarities agree with the observed similar kinetic behaviors. However, some differences can be identified in the orientation of some of the loops, such as 1, 2, and 4, which can be responsible for the significantly different *K*_I_ values or activation free energy barriers measured and computed, respectively, for the inhibition of cruzain and cathepsin L by compound **11**.

**Figure 7:**
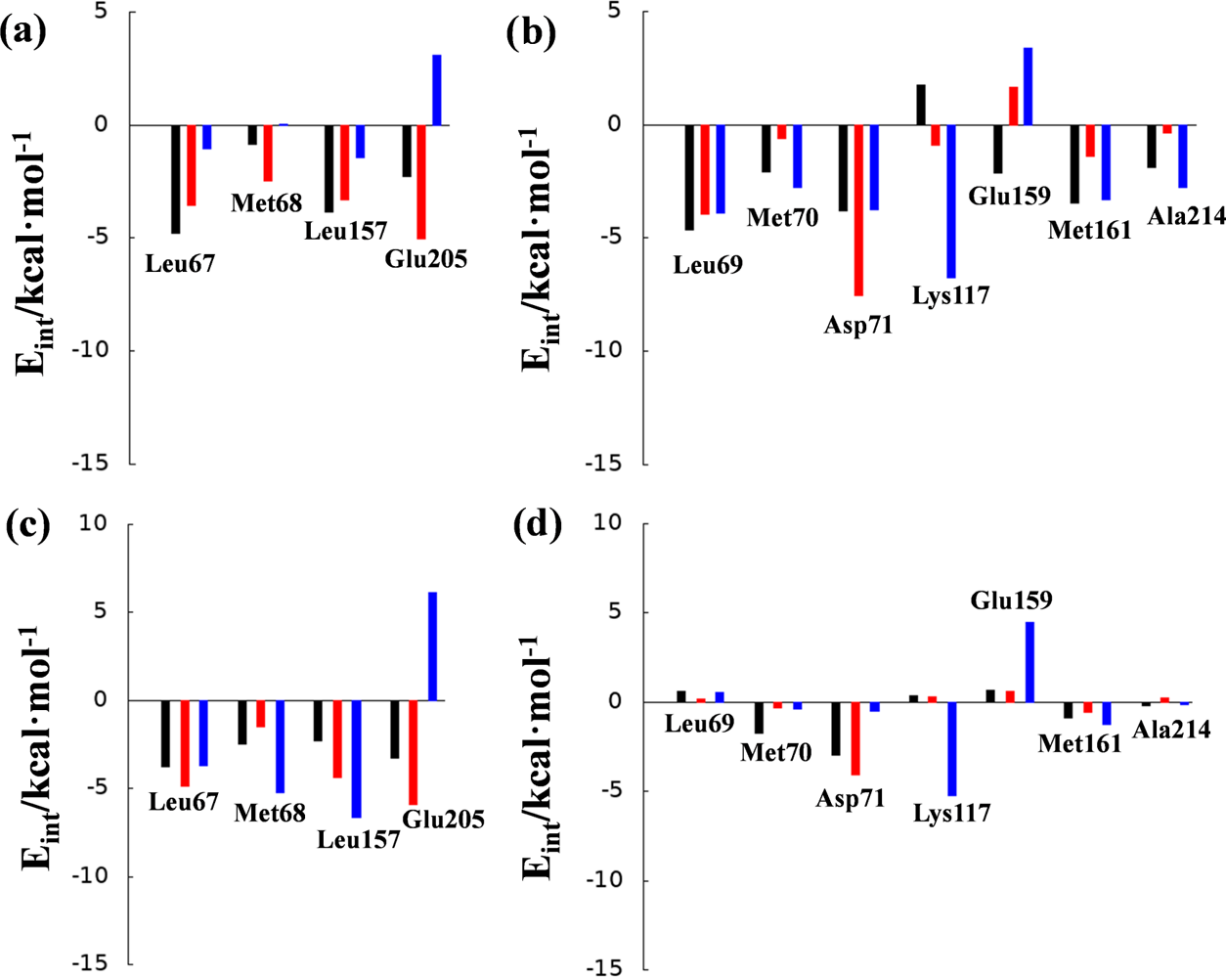
Average interaction energies (electrostatic plus Lennard–Jones) between the P2 position of the inhibitor and amino acids of the S2 pocket of cysteine proteases cruzain (a and c) and cathepsin L (b and d). Panels (a) and (b) show the results computed in the **E·I** reactant complex, while panels (c) and (d) show the results computed in the **E-I** covalent adduct. The black bars correspond to the values of inhibitor **11**,^35^ the red bars correspond to the values of inhibitor **11a**, and the blue bars correspond to the values of inhibitor **11b**.

Figure 7 shows the interactions established between the P2 position of the three inhibitors and the amino acids of the S2 pocket of cruzain and cathepsin L, while the QM/MM interaction energies (electrostatic plus Lennard–Jones) between the P2 position of the inhibitors and amino acids of the S2 pocket of the two CPs were computed as an average over 10,000 structures from the AM1d/MM MD simulations in the initial **E·I** reactant complex and the **E-I** covalent adduct. The most important interactions, larger than 1 kcal·mol^–1^, computed in the **E·I** reactant complex state of cruzain and cathepsin L inhibition processes are shown in Figure 7a,b, respectively, while the corresponding ones computed in the **E·I** covalent adduct are shown in Figure 7c,d, respectively (see Figures S8–S11 of the Supporting Information for the representations of the full list of interactions).

Analysis of the interactions in the **E·I** reactant complexes shows that in the inhibition process by **11a**, replacement of the Phe residue of compound **11** with Trp provokes an increase in favorable interactions with the S2 pocket of cruzain (red bars vs black bars in Figure 7a), basically due to the interactions with residues Met68 (1.6 kcal·mol^–1^) and Glu205 (2.7 kcal·mol^–1^). The interaction between the Trp moiety of the inhibitor and the remaining residues of the S2 pocket of cruzain (Leu67 and Leu157) does not appear to be significantly different from that established with the Phe moiety of compound **11**. On the contrary, the limited activity of **11b** against cruzain could be related to the unfavorable interaction with Glu205 of the S2 pocket of the enzyme (blue bars in Figure 7a), 3.1 kcal·mol^–1^, a residue that plays an important role in recognition during the inhibition mechanism of cruzain.^35,47,52^ In addition, there are less favorable interactions with the rest of the key residues than those in the case of inhibitor **11** (blue bars vs black bars in Figure 7a). In the case of cathepsin L, several residues of the S2 pocket interact in a different manner with the P2 position of inhibitors **11**, **11a**, and **11b** in the **E·I** reactant complex (see Figure 7b). Thus, the favorable interactions with Leu69, Met70, Met161, and Ala214 are reduced when comparing **11** and **11a**, while the originally unfavorable interaction with Lys117 in **11** becomes a favorable interaction in **11a**. However, the most significant change occurs in the interactions with residues Asp71 and Glu159: while the favorable interaction of **11** with Glu159 (−2.1 kcal·mol^–1^) becomes unfavorable with Trp in **11a** (1.7 kcal·mol^–1^), the new Trp:Asp71 interaction in **11a** (−7.5 kcal·mol^–1^) is considerably more favorable than the Phe:Asp71 interaction in **11** (−3.8 kcal·mol^–1^). The interactions between P2 of **11b** and S2 of cathepsin L (blue bars in Figure 7b) are similar to those detected in **11a**, except for those involving residues Asp71 and Lys117. The interaction with Asp71 is, similar to that in **11**, significantly less favorable than that in **11a**, while the interaction with Lys117 in **11b** becomes more favorable than that in **11a** (−6.8 kcal·mol^–1^ in **11b** vs −0.9 kcal·mol^–1^ in **11a**). It is also remarkable how the interaction between the Glu159 residue and the 4-NO_2_–Phe fragment of **11b** (3.4 kcal·mol^–1^) is more unfavorable than that in **11a**.

The interactions established between the P2 position of the inhibitors and the amino acids of the S2 pocket of cruzain and cathepsin L computed in the E-I covalent adduct (Figure 7c,d) coincide roughly with the values obtained in the E·I reactant complex. Thus, the pattern of interactions does not qualitatively change along the reaction, which is reasonable, taking into account that the P2 moiety of the inhibitor is not being modified when the enzyme–inhibitor covalent bond forms. According to the analysis of the pose of the compounds in the active site of the enzyme and the global pattern of protein–inhibitor interactions computed in both the initial and final states of the inhibition processes, it is possible to rationalize the trends in the activation and reaction energies of **11a** with respect to those of **11b** in both enzymes, in qualitative agreement with the experimental data, despite the almost negligible differences. The polar repulsion interactions between the nitro group of the 4-nitrophenyl alanine residue in **11b** and polar glutamic groups at the P2 site in both cruzain and cathepsin L (Glu205 and Glu159, respectively) also afford a rational explanation for the lower activity of **11b**.

However, according to our results, it is likely that these compounds, and in particular **11a** and **11b** that do not show significant specificity for cruzain or cathepsin L, will inhibit other CPs, which cannot be a good solution because of the possible side effects in future possible medical treatments. However, the deep comparative analysis of the inhibition of both enzymes with the three tested compounds can be used to distinguish and guide the design of selective CP inhibitors. In this regard, for instance, compound **11** clearly shows better inhibitory activity for cruzain than cathepsin L, while **11a** and **11b** show indiscernible activities.

In all, analysis of structures of cruzain and cathepsin L and the interactions of the three inhibitors in the **E·I** reactant complex and in the **E-I** covalent adduct suggests that the role of residues Lys117, Asp71, and Glu159 could be important for the inhibition process of cathepsin L, while Glu205 could be decisive for the inhibitioin of cruzain.

## Conclusions

A study of the influence of the P2 site residue on dipeptidyl nitroalkene inhibitors has been carried out in a combined theoretical and experimental study. Two new inhibitors having a tryptophan (**11a**) or a 4-nitrophenyl alanine (**11b**) moiety at the P2 site were proposed, computationally studied, synthesized, and tested in vitro for two cysteine proteases: cruzain and cathepsin L. By comparison with the original inhibitor having a phenyl alanine, some influence of the chemical groups at the P2 site is observed experimentally, and the results are rationalized in accordance with our computational study. The mechanism of the reaction of **E-I** covalent complex formation was studied by generating the QM/MM free energy surfaces of the chemical steps for both inhibitors. Two new proposed dipeptidyl nitroalkenes **11a** and **11b** show better kinetic and thermodynamic values for the inhibition of cathepsin L than original compound **11**. This computational prediction is in qualitative agreement with our experimental determination of in vitro *K*_I_ values, despite our simulations being focused on the chemical step (from **E·I** to **E-I**), and *K*_I_ values are a measure of the full process from the solvent-separated species **E** + **I**. Regarding the inhibition of cruzain CP, the three tested compounds show almost indistinguishable inhibition activity. According to our results, **11a** and **11b** do not show significant specificity for cruzain or cathepsin L, and consequently, it is likely that they will inhibit other CPs, which cannot be a good solution because of the possible side effects in the future possible medical treatments. In contrast, compound **11** would present a measurable selectivity on cruzain inhibition. However, the analysis derived from this study suggests that the proposed dipeptidyl nitroalkene compounds can be used to guide the (re)design of selective CP inhibitors and, in particular, the one with a bulky Trp group at the P2 site, **11a**, that shows promising *in vitro* reversible covalent inhibition activities against cruzain and especially against cathepsin L. Analysis of the interactions established between the P2 site of the inhibitors and the S2 pocket of the two studied CPs suggests that these particular residues of the active site that can be the target to improve future designs: Lys117, Asp71, and Glu159 could be important for the inhibition process of cathepsin L, and Glu205 could be important in the case of the inhibition of cruzain.

## Supporting Information Available

RMSD computed along the classical MD simulation for the backbone atoms of the protein; force field parameters for inhibitors; details of the active site and QM-MM partitioning; M06-2X/6-31+G(d,p)/MM FESs obtained with umbrella sampling and protein–inhibitor nonbonding interaction energies; description of ^1^H NMR, ^13^C NMR, IR, and HRMS spectra; and copy of ^1^H NMR and ^13^C NMR spectra for all compounds (PDF)
