# Supplementary material for: Impact of the Recognition Part of Dipeptidyl Nitroalkene Compounds on the Inhibition Mechanism of Cysteine Proteases Cruzain and Cathepsin L
Source: ACS Catal. 2023 Apr 24;13(9):6289–300. doi: 10.1021/acscatal.3c01035 (PMC10167892; doi:10.1021/acscatal.3c01035)
Supplement: Supplementary file 1 — cs3c01035_si_001.pdf [file cs3c01035_si_001.pdf]

Supporting Information

Impact of the Recognition Part of Dipeptidyl Nitroalkene Compounds on the Inhibition Mechanism of Cysteine Proteases Cruzain and Cathepsin L.

Kemel Arafet,[1,2] Santiago Royo,[3] Tanja Schirmeister,[4] Fabian Barthels, [4] Florenci V. González,[3,] Vicent Moliner[2,*]*

1. Dipartimento di Scienze degli Alimenti e del Farmaco, Università degli Studi di Parma, Italy.
2. BioComp Group, Institute of Advanced Materials (INAM), Universitat Jaume I, 12071 Castelló, Spain.
3. Departament de Química Inorgànica i Orgànica, Universitat Jaume I, 12071 Castelló, Spain.
4. Institute of Pharmaceutical and Biomedical Sciences, Johannes Gutenberg-Universität, 55128 Mainz, Germany.

Corresponding Authors:

*V. M., e-mail address: moliner@uji.es; tel, +34964728084.

*F. V. G., e-mail address: fgonzale@uji.es

Contents

I- Computational Methods……………………………………………………………S2

II- Computational Results…………………...…………………………………………..S4

III-Characterization data for synthesized compounds……………………………..S22

IV- ^{1}H and ^{13}C-NMR Spectra……………………………………….……………….S24

V-References….……………………………………...………………………S28

I- Computational Methods

Models set up. The cruzain-inhibitor molecular models were constructed from the X-ray crystal structure of cruzain from *Trypanosoma cruzi* with PDB code 1AIM[1] and 2.0 Å resolution, that contains Cbz-Tyr-Ala-CH_2F covalently bound to the active site. The inhibitor was replaced by the nitroalkene inhibitors **11a** and **11b**. The initial coordinates for building the cathepsin L-inhibitor models were taken from the X-ray crystal structure of the human cathepsin L with PDB code 2XU3[2] by replacing the nitrile ((2S,4R)-4-(2-chlorophenyl)sulfonyl-1-[1-(5-chlorothiophen-2-yl)cyclopropyl]carbonyl-N-[1-(iminomethyl)cyclopropyl]pyrrolidine-2-carboxamide) inhibitor by the nitroalkene inhibitors **11a** and **11b**. These X-ray structures were already used in our laboratory to carry out different computational studies related to the CPs.[3-7]

In all molecular models, the missing hydrogen atoms of the X-ray structure were added at pH 7 using the tLEaP module of Amber Tools program[8] within the pKa values of the titratable residues previously calculated within the empirical PROPKA 3.1 program.[9] Missing force field parameters for the inhibitors were computed using Antechamber program[10] available in AmberTools package (see Tables S1 and S2). A total of 7 and 8 Na^+ counterions were added for cruzain and cathepsin L models, respectively. Finally, the systems were solvated in orthorhombic boxes of TIP3P[11] water molecules with the following sizes: cruzain 69.5 Å x 71.5 Å x 79.8 Å and cathepsin L 69.8 Å x 80.7 Å x 80.4 Å. The next step for each model consisted of 10^5 steps of conjugate-gradient minimization, followed by series of molecular dynamics (MD) in the NVT ensemble with the AMBER ff03 force field,[12] as implemented in NAMD software:[13] 100 initial ps for heating up to 310 K, followed by 5 ns of equilibration, using the Langevin thermostat.[14] All simulations made use of the PME algorithm for accounting of the electrostatic interactions with a force-switch scheme ranging from 14.5 to 16 Å, and a time step of 1 fs. Analysis of the time evolution of the root-mean-square deviation of the backbone atoms of the protein models confirms that all systems were equilibrated (see Figures S1 and S2 in the Supporting Information). Analysis was done using cpptraj facility.[15]

QM/MM Simulations. In this work, an additive hybrid QM/MM scheme was employed for the construction of the total Hamiltonian where the total energy is obtained from the sum of each contribution to the energy. The QM region was initially described with the AM1d semiempirical Hamiltonian,[16] and contains the full inhibitor, the residue Cys25, the imidazole ring of His159 (His 163 in the case of the cathepsin L cysteine protease, see Figure S3). AM1d semiempirical Hamiltonian has been employed in different

computational studies related to the mechanisms of catalysis and inhibition of cysteine proteases.[3-7,17,18] The rest of the system, protein and water molecules, were described by the OPLS-AA[19] and TIP3P[11] force fields, respectively. Hydrogen link atoms were used to saturate the valence of the QM-MM frontier bonds.[20] All residues further than 25 Å from the C_β atom of the inhibitor were kept frozen during the simulations. A force switching function with a cutoff distance in the range of 14.5 to 16 Å and periodic boundary conditions were employed was used to treat the non-bonding interactions. Contribution of each residue of the protein to the interaction energy with defined part of substrate was computer using the following expression:

$$E_{QM/MM}^{Int} = \sum \left\langle \Psi \left| \frac{q_{MM}}{r_{e,MM}} \right| \Psi \right\rangle + \sum\sum \frac{Z_{QM} q_{MM}}{r_{QM,MM}} + E_{QM/MM}^{vdW} \qquad (1)$$

This interaction energy can be exactly decomposed in a sum over residues provided that the polarized wave function (Ψ) is employed to evaluate this energy contribution. The global polarization effect can be obtained from the gas phase energy difference between the polarized, Ψ, and non-polarized, Ψ_0, wave functions.

Potential Energy Surfaces. Potential energy surfaces (PESs) were obtained by grid scanning of distinguished reaction coordinates most suitable for describing each of the chemical step, as described below. A harmonic constraint was used to maintain the proper interatomic distances along the reaction coordinate, and a series of conjugate gradient optimizations and L-BFGS-B optimization algorithms were applied to obtain the final potential energy of the minimized constrained geometry. A micro-macro iteration optimization algorithm[21,22] was used to localize, optimize, and characterize the TS structures using a Hessian matrix containing all the coordinates of the QM subsystem at M06-2X/MM level of theory with the standard 6-31+G(d,p) basis set. The gradient norm of the remaining movable atoms was maintained less than 0.01 kcal·mol^{-1}·Å^{-1}. Intrinsic reaction coordinates (IRCs) were traced down from located TSs to the valleys of the reactants, intermediates, and products in mass-weighted Cartesian coordinates.

Free Energy Surfaces. Free energy surfaces (FESs), in terms of potential of mean force (PMF), were generated using umbrella sampling (US) and the weighted histogram analysis method (WHAM) to recover the probabilities.[23,24] The error associated to this method, when properly carried out, is usually accepted to be around 1 kcal·mol^{-1}.[25] The harmonic umbrella sampling force constants was 2500 kJ·mol^{-1}·Å^{-2}. 20 ps of equilibration

and 40 ps of production, with a time step of 1 fs, were used in every window of the PMFs. Based on the information derived from the previously computed PESs, monodimensional PMF (1D-PMF) at AM1d/MM level was generated with the bond-forming distance, d(SG-C_β) as ζ for the study of the sulfur attack on the C_β. This first step leads to an intermediate called INT-C_β. For the second step, the protonation of the INT-C_β intermediate, a 1D-PMF AM1d/MM was generated using as ζ the antisymmetric combination of two distances defined for the hydrogen transfer from the His159 to the C_α, d(N3-H3)-d(C_α-H3). See Figures S4-S7. The first step of the inhibition by **11a** required series of 34 and 44 simulation windows for cruzain and cathepsin L, respectively. The second step required series of 81 and 81 simulation windows for cruzain and cathepsin L, respectively. The first of the inhibition by **11b** required series of 49 and 71 simulation windows for cruzain and cathepsin L, respectively. The second step required series of 59 and 181 simulation windows for cruzain and cathepsin L, respectively.

Spline corrections. A correction term was interpolated to any value along the reaction coordinates in the AM1d/MM FES. A continuous energy function is used to obtain the corrected PMFs:

$$E = E_{LL/MM} + S\left[\Delta E_{LL}^{HL}(\xi)\right] \quad (2)$$

where S denotes a spline function and is the difference between the energies obtained at low-level (LL) and high-level (HL) of theory of the QM part. The correction term is expressed as a function of the distinguished reaction coordinate, ζ. The AM1d semiempirical Hamiltonian[16] was used as LL method, while a density functional theory (DFT)-based method was selected for the HL energy calculation. In particular, the hybrid M06-2X functional[26] with the standard 6-31+G(d,p) basis set,[27] following Truhlar and co-workers suggestions,[26,28] was used employing the *Gaussian09* program,[29] combined with the fDYNAMO library.[30]

II- Computational Results

Table S1. Atom types, charges and parameters obtained for the inhibitor **11a** generated on the **E·I** reactant complex using antechamber package included in AmberTools.

Atom name	Atom type	Charge	Parameters
CB	c3	-0.011200	NONBON
CG	cc	-0.165400	ca 1.9080 0.0860
CD1	cd	-0.099100	ha 1.4590 0.0150

CD2	ca	-0.088800
CE2	ca	-0.046200
CE3	ca	-0.086000
NE1	na	-0.188400
CZ2	ca	-0.142000
CZ3	ca	-0.150000
CH2	ca	-0.110000
C1	ca	-0.126300
N1	n	-0.513900
O1	os	-0.434900
C2	ca	-0.107500
N02	no	0.275100
N2	n	-0.564900
O2	o	-0.603000
C3	ca	-0.134000
O03	o	-0.214500
O3	o	-0.621100
C4	ca	-0.120000
O04	o	-0.214500
C5	ca	-0.134000
C6	ca	-0.107500
C7	c3	0.191700
C8	c	0.736100
C9	c3	0.038700
C17	c	0.654100
C18	c3	0.129900
C19	c3	-0.110100
Cβ	c2	-0.096200
Cα	c2	-0.218100
HD1	h4	0.168000
HE1	hn	0.301700
HE3	ha	0.134000
HZ2	ha	0.134000
HZ3	ha	0.134000
HH2	ha	0.132000
H01	hc	0.053033
H02	hc	0.053033
H03	hc	0.053033
H04	h4	0.209000
H05	ha	0.162000
H06	hn	0.330500
H07	h1	0.084700
H09	hn	0.348500
H10	hc	0.079200
H11	hc	0.079200
H16	ha	0.138000
H17	h1	0.079200
H28	h1	0.089700
H31	ha	0.138000
H32	ha	0.134000
H33	ha	0.133000
H34	ha	0.134000
H36	h1	0.079200

c3	1.9080	0.1094
h1	1.3870	0.0157
os	1.6837	0.1700
c	1.9080	0.0860
o	1.6612	0.2100
n	1.8240	0.1700
hn	0.6000	0.0157
hc	1.4870	0.0157
c2	1.9080	0.0860
no	1.8240	0.1700
h4	1.4090	0.0150
cc	1.9080	0.0860
cd	1.9080	0.0860
na	1.8240	0.1700

BOND

ca-ca	461.10	1.398
ca-ha	345.80	1.086
ca-c3	321.00	1.516
c3-h1	330.60	1.097
c3-os	308.60	1.432
os-c	390.80	1.358
c -o	637.70	1.218
c -n	427.60	1.379
n -hn	403.20	1.013
n -c3	328.70	1.462
c3-c	313.00	1.524
c3-c3	300.90	1.538
c3-c2	326.80	1.510
c3-hc	330.60	1.097
c2-c2	569.40	1.334
c2-ha	343.10	1.088
c2-no	343.00	1.448
c2-h4	344.60	1.087
no-o	741.80	1.226
c3-cc	334.80	1.502
cc-cd	500.90	1.373
cc-ca	385.10	1.456
cd-h4	352.00	1.082
cd-na	425.80	1.380
na-hn	408.40	1.010
na-ca	420.50	1.384

ANGLE

ca-ca-ha	48.180	119.880
ca-ca-ca	66.620	120.020
ca-ca-c3	63.530	120.770
ca-c3-h1	46.990	109.560
ca-c3-os	68.260	108.950
c3-os-c	63.280	115.980
h1-c3-h1	39.240	108.460
h1-c3-os	50.800	109.780
os-c -o	75.320	123.250
os-c -n	75.320	109.220
c -n -hn	48.330	117.550

Parameters

ANGLE

c -n -c3	63.390	120.690
o -c -n	74.220	123.050
n -c3-c	67.000	109.060
n -c3-h1	49.840	108.880
n -c3-c3	65.910	111.610
hn-n -c3	45.800	117.680
c3-c -n	66.790	115.180
c3-c -o	67.400	123.200

DIHEDRALS

c3-c3-cc-cd	1	0.000	0.000	3.000
c3-c3-cc-ca	1	0.000	0.000	3.000
c -c3-c3-hc	1	0.156	0.000	3.000
c -c3-c3-cc	1	0.156	0.000	3.000
c -n -c3-c2	1	0.000	0.000	2.000
n -c -c3-h1	1	0.000	180.000	2.000
n -c -c3-c3	1	0.100	0.000	-4.000
n -c -c3-c3	1	0.070	0.000	2.000

c3-c3-hc	46.340	109.800
c3-c3-cc	63.470	111.930
c -c3-h1	47.040	108.220
c -c3-c3	63.270	111.040
n -c3-c2	66.680	111.290
c3-c2-c2	64.060	123.630
c3-c2-ha	45.940	115.680
c3-c3-c2	63.410	111.560
c3-c3-h1	46.390	109.560
hc-c3-hc	39.400	107.580
c2-c3-h1	47.070	109.960
c2-c2-no	67.410	123.460
c2-c2-h4	49.400	122.670
c2-c2-ha	49.850	120.430
c2-no-o	69.430	117.670
no-c2-h4	49.300	113.380
o -no-o	76.730	125.080
c3-cc-cd	64.710	119.450
c3-cc-ca	61.340	126.520
hc-c3-cc	47.180	110.490
cc-cd-h4	47.270	128.480
cc-cd-na	73.430	106.990
cc-ca-ca	65.020	120.790
cd-cc-ca	67.620	113.510
cd-na-hn	46.750	125.500
cd-na-ca	67.400	113.150
h4-cd-na	49.790	120.530
na-ca-ca	69.080	118.340
hn-na-ca	46.630	125.540

IMPROPER

ca-ca-ca-ha	1.1	180.0	2.0
c3-ca-ca-ca	1.1	180.0	2.0
n -o -c -os	10.5	180.0	2.0
c -c3-n -hn	1.1	180.0	2.0
c3-n -c -o	10.5	180.0	2.0
c2-c3-c2-ha	1.1	180.0	2.0
c2-h4-c2-no	1.1	180.0	2.0
c3-ca-cc-cd	1.1	180.0	2.0
cc-h4-cd-na	1.1	180.0	2.0
ca-cd-na-hn	1.1	180.0	2.0
ca-ca-ca-na	1.1	180.0	2.0
ca-ca-ca-cc	1.1	180.0	2.0

DIHEDRALS

ca-ca-ca-ca	1	3.625	180.000	2.000
ca-ca-ca-c3	1	3.625	180.000	2.000
ca-ca-ca-ha	1	3.625	180.000	2.000
ca-ca-c3-h1	1	0.000	0.000	2.000
ca-ca-c3-os	1	0.000	0.000	2.000
ha-ca-ca-ha	1	3.625	180.000	2.000
ha-ca-ca-c3	1	3.625	180.000	2.000
ca-c3-os-c	1	0.383	0.000	3.000
c3-os-c -o	1	2.700	180.000	-2.000
c3-os-c -o	1	1.400	180.000	1.000
c3-os-c -n	1	2.700	180.000	2.000
h1-c3-os-c	1	0.383	0.000	3.000
os-c -n -hn	1	2.500	180.000	2.000
os-c -n -c3	1	2.500	180.000	2.000
c3-c -n -hn	1	2.500	180.000	2.000
n -c3-c2-c2	1	0.000	0.000	2.000
n -c3-c2-ha	1	0.000	0.000	2.000
c3-c2-c2-no	1	6.650	180.000	2.000
c3-c2-c2-h4	1	6.650	180.000	2.000
c3-c3-c2-c2	1	0.000	0.000	2.000
c3-c3-c2-ha	1	0.000	0.000	2.000
hc-c3-c3-c2	1	0.156	0.000	3.000
hc-c3-c3-h1	1	0.156	0.000	3.000
c2-c3-n -hn	1	0.000	0.000	2.000
c2-c2-no-o	1	0.750	180.000	2.000
c2-c2-c3-h1	1	0.000	0.000	2.000
no-c2-c2-ha	1	6.650	180.000	2.000
o -no-c2-h4	1	0.750	180.000	2.000
h4-c2-c2-ha	1	6.650	180.000	2.000
ha-c2-c3-h1	1	0.000	0.000	2.000
o -c -c3-h1	1	0.800	0.000	-1.000
o -c -c3-h1	1	0.000	0.000	-2.000
o -c -c3-h1	1	0.080	180.000	3.000
o -c -c3-c3	1	0.000	180.000	2.000
h1-c3-c3-cc	1	0.156	0.000	3.000
c3-cc-cd-h4	1	4.000	180.000	2.000
c3-cc-cd-na	1	4.000	180.000	2.000
c3-cc-ca-ca	1	0.700	180.000	2.000
hc-c3-cc-cd	1	0.000	0.000	3.000
hc-c3-cc-ca	1	0.000	0.000	3.000
cc-cd-na-hn	1	1.700	180.000	2.000
cc-cd-na-ca	1	1.700	180.000	2.000
cc-ca-ca-na	1	3.625	180.000	2.000
cc-ca-ca-ca	1	3.625	180.000	2.000
cc-ca-ca-ha	1	3.625	180.000	2.000
cd-cc-ca-ca	1	0.700	180.000	2.000
cd-na-ca-ca	1	0.300	180.000	2.000
h4-cd-cc-ca	1	4.000	180.000	2.000
h4-cd-na-hn	1	1.700	180.000	2.000
h4-cd-na-ca	1	1.700	180.000	2.000
na-cd-cc-ca	1	4.000	180.000	2.000
na-ca-ca-ca	1	3.625	180.000	2.000
na-ca-ca-ha	1	3.625	180.000	2.000
hn-na-ca-ca	1	0.300	180.000	2.000
c -n -c3-c	1	0.850	180.000	-2.000
c -n -c3-c	1	0.800	0.000	1.000
c -n -c3-h1	1	0.000	0.000	2.000
c -n -c3-c3	1	0.500	180.000	-4.000
c -n -c3-c3	1	0.150	180.000	-3.000
c -n -c3-c3	1	0.000	0.000	-2.000
c -n -c3-c3	1	0.530	0.000	1.000
o -c -n -hn	1	2.500	180.000	-2.000
o -c -n -hn	1	2.000	0.000	1.000
o -c -n -c3	1	2.500	180.000	2.000
n -c3-c -n	1	1.700	180.000	-1.000
n -c3-c -n	1	2.000	180.000	2.000
n -c3-c -o	1	0.000	180.000	2.000
n -c3-c3-hc	1	0.156	0.000	3.000
n -c3-c3-cc	1	0.156	0.000	3.000
hn-n -c3-c	1	0.000	0.000	2.000
hn-n -c3-h1	1	0.000	0.000	2.000
hn-n -c3-c3	1	0.000	0.000	2.000
c3-c -n -c3	1	0.000	0.000	-2.000
c3-c -n -c3	1	1.500	180.000	1.000

Table S2. Atom types, charges and parameters obtained for the inhibitor **11b** generated on the **E·I** reactant complex using antechamber package included in AmberTools.

Atom name	Atom type	Charge
C1	ca	-0.104300
N01	no	0.313200
N1	n	-0.541900
O01	o	-0.204500
O1	os	-0.430900
C2	ca	-0.108000
N02	no	0.275100
N2	n	-0.561900
O2	o	-0.607000
C3	ca	-0.131000
O03	o	-0.211500
O3	o	-0.617100
C4	ca	-0.118000
O04	o	-0.211500
C5	ca	-0.131000
O05	o	-0.204500
C6	ca	-0.108000
C7	c3	0.199700
C8	c	0.742100
C9	c3	0.053700
C10	c3	-0.056100
C11	ca	-0.049300
C12	ca	-0.139000
C13	ca	-0.062000
C14	ca	-0.175200
C15	ca	-0.062000
C16	ca	-0.139000
C17	c	0.648100
C18	c3	0.129900
C19	c3	-0.109100
Cβ	c2	-0.100200
Cα	c2	-0.217100
H01	hc	0.054367
H02	hc	0.054367
H03	hc	0.054367
H04	h4	0.207000
H05	ha	0.165000
H06	hn	0.338500
H09	hn	0.348500
H16	ha	0.137000
H17	h1	0.065200
H19	h1	0.086700
H20	hc	0.090200
H22	ha	0.150000
H23	ha	0.174500
H25	ha	0.174500
H28	h1	0.091700
H31	ha	0.137000
H32	ha	0.136500
H33	ha	0.135000
H34	ha	0.136500
H36	h1	0.065200
H37	hc	0.090200
H42	ha	0.150000

Parameters

NONBON

o	1.6612	0.2100
no	1.8240	0.1700
ca	1.9080	0.0860
ha	1.4590	0.0150
c3	1.9080	0.1094
hc	1.4870	0.0157
c	1.9080	0.0860
n	1.8240	0.1700
c2	1.9080	0.0860
h4	1.4090	0.0150
h1	1.3870	0.0157
hn	0.6000	0.0157
os	1.6837	0.1700

BOND

o -no	741.80	1.226
no-ca	321.70	1.469
ca-ca	461.10	1.398
ca-ha	345.80	1.086
ca-c3	321.00	1.516
c3-hc	330.60	1.097
c3-c3	300.90	1.538
c3-c	313.00	1.524
c3-h1	330.60	1.097
c3-n	328.70	1.462
c -n	427.60	1.379
c -o	637.70	1.218
n -hn	403.20	1.013
c3-c2	326.80	1.510
c2-c2	569.40	1.334
c2-ha	343.10	1.088
c2-no	343.00	1.448
c2-h4	344.60	1.087
c -os	390.80	1.358
os-c3	308.60	1.432

ANGLE

o -no-o	76.730	125.080
o -no-ca	68.700	117.760
no-ca-ca	66.770	119.010
ca-ca-ca	66.620	120.020
ca-ca-ha	48.180	119.880
ca-ca-c3	63.530	120.770
ca-c3-hc	46.800	110.470
ca-c3-c3	63.150	112.070
c3-c3-c	63.270	111.040
c3-c3-h1	46.390	109.560
c3-c3-n	65.910	111.610
hc-c3-hc	39.400	107.580
hc-c3-c3	46.340	109.800
c3-c -n	66.790	115.180
c3-c -o	67.400	123.200
c3-n -hn	45.800	117.680
c3-n -c	63.390	120.690
c -c3-h1	47.040	108.220
c -c3-n	67.000	109.060
c -n -hn	48.330	117.550
n -c -o	74.220	123.050
n -c3-c2	66.680	111.290

			n -c3-h1 49.840 108.880
			c3-c2-c2 64.060 123.630
			c3-c2-ha 45.940 115.680
			c3-c3-c2 63.410 111.560
			c2-c3-h1 47.070 109.960
			c2-c2-no 67.410 123.460
			c2-c2-h4 49.400 122.670
			c2-c2-ha 49.850 120.430
			c2-no-o 69.430 117.670
			no-c2-h4 49.300 113.380
			n -c -os 75.320 109.220
			c -os-c3 63.280 115.980
			o -c -os 75.320 123.250
			os-c3-h1 50.800 109.780
			os-c3-ca 68.260 108.950
			h1-c3-h1 39.240 108.460
			h1-c3-ca 46.990 109.560

Parameters

IMPROPER

ca-o -no-o	1.1	180.0	2.0
ca-ca-ca-no	1.1	180.0	2.0
ca-ca-ca-ha	1.1	180.0	2.0
c3-ca-ca-ca	1.1	180.0	2.0
c3-n -c -o	10.5	180.0	2.0
c -c3-n -hn	1.1	180.0	2.0
c2-c3-c2-ha	1.1	180.0	2.0
c2-h4-c2-no	1.1	180.0	2.0
n -o -c -os	10.5	180.0	2.0

DIHEDRALS

o -no-ca-ca	1	0.600	180.000	2.000
no-ca-ca-ca	1	3.625	180.000	2.000
no-ca-ca-ha	1	3.625	180.000	2.000
ca-ca-ca-ha	1	3.625	180.000	2.000
ca-ca-ca-ca	1	3.625	180.000	2.000
ca-ca-ca-c3	1	3.625	180.000	2.000
ca-ca-c3-hc	1	0.000	0.000	2.000
ca-ca-c3-c3	1	0.000	0.000	2.000
ha-ca-ca-ha	1	3.625	180.000	2.000
ha-ca-ca-c3	1	3.625	180.000	2.000
ca-c3-c3-c	1	0.156	0.000	3.000
ca-c3-c3-h1	1	0.156	0.000	3.000
ca-c3-c3-n	1	0.156	0.000	3.000
c3-c3-c -n	1	0.100	0.000	-4.000
c3-c3-c -n	1	0.070	0.000	2.000
c3-c3-c -o	1	0.000	180.000	2.000
c3-c3-n -hn	1	0.000	0.000	2.000
c3-c3-n -c	1	0.500	180.000	-4.000
c3-c3-n -c	1	0.150	180.000	-3.000
c3-c3-n -c	1	0.000	0.000	-2.000
c3-c3-n -c	1	0.530	0.000	1.000
hc-c3-c3-c	1	0.156	0.000	3.000
hc-c3-c3-h1	1	0.156	0.000	3.000
hc-c3-c3-n	1	0.156	0.000	3.000
c3-c -n -c3	1	0.000	0.000	-2.000
c3-c -n -c3	1	1.500	180.000	1.000
c3-c -n -hn	1	2.500	180.000	2.000
c3-n -c -o	1	2.500	180.000	2.000

DIHEDRALS

c3-n -c -os	1	2.500	180.000	2.000
c -c3-n -hn	1	0.000	0.000	2.000
c -c3-n -c	1	0.850	180.000	-2.000
c -c3-n -c	1	0.800	0.000	1.000
c -n -c3-c2	1	0.000	0.000	2.000
c -n -c3-h1	1	0.000	0.000	2.000
n -c -c3-h1	1	0.000	180.000	2.000
n -c -c3-n	1	1.700	180.000	-1.000
n -c -c3-n	1	2.000	180.000	2.000
n -c3-c2-c2	1	0.000	0.000	2.000
n -c3-c2-ha	1	0.000	0.000	2.000
c3-c2-c2-no	1	6.650	180.000	2.000
c3-c2-c2-h4	1	6.650	180.000	2.000
c3-c3-c2-c2	1	0.000	0.000	2.000
c3-c3-c2-ha	1	0.000	0.000	2.000
hc-c3-c3-c2	1	0.156	0.000	3.000
c2-c3-n -hn	1	0.000	0.000	2.000
c2-c2-no-o	1	0.750	180.000	2.000
c2-c2-c3-h1	1	0.000	0.000	2.000
no-c2-c2-ha	1	6.650	180.000	2.000
o -no-c2-h4	1	0.750	180.000	2.000
h4-c2-c2-ha	1	6.650	180.000	2.000
ha-c2-c3-h1	1	0.000	0.000	2.000
h1-c3-n -hn	1	0.000	0.000	2.000
hn-n -c -o	1	2.500	180.000	-2.000
hn-n -c -o	1	2.000	0.000	1.000
o -c -c3-h1	1	0.800	0.000	-1.000
o -c -c3-h1	1	0.000	0.000	-2.000
o -c -c3-h1	1	0.080	180.000	3.000
o -c -c3-n	1	0.000	180.000	2.000
n -c -os-c3	1	2.700	180.000	2.000
hn-n -c -os	1	2.500	180.000	2.000
c -os-c3-h1	1	0.383	0.000	3.000
c -os-c3-ca	1	0.383	0.000	3.000
o -c -os-c3	1	2.700	180.000	-2.000
o -c -os-c3	1	1.400	180.000	1.000
os-c3-ca-ca	1	0.000	0.000	2.000
h1-c3-ca-ca	1	0.000	0.000	2.000

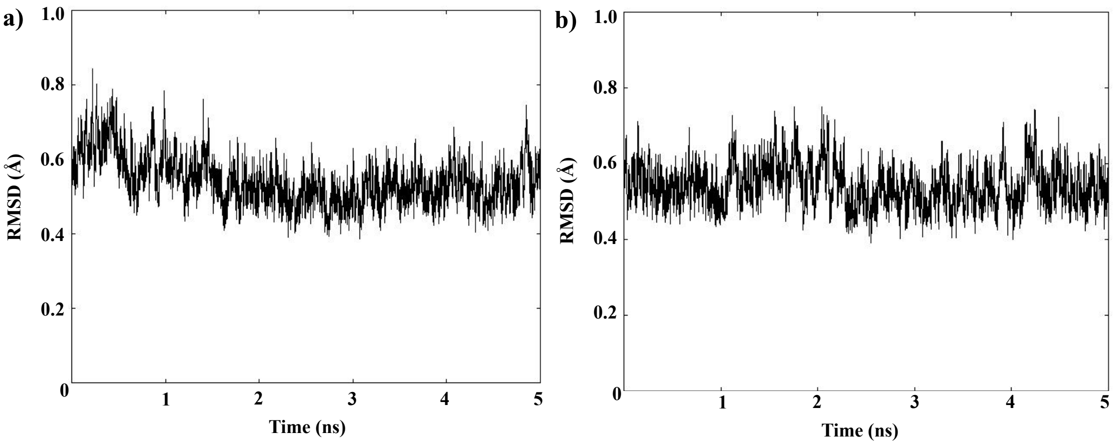

Figure S1. RMSD along the classical MD simulation for the backbone atoms of the cruzain cysteine protease. Simulations performed on the non-covalent reactant state corresponding to the **E·I** reactant complex. a) **11a**. b) **11b**.

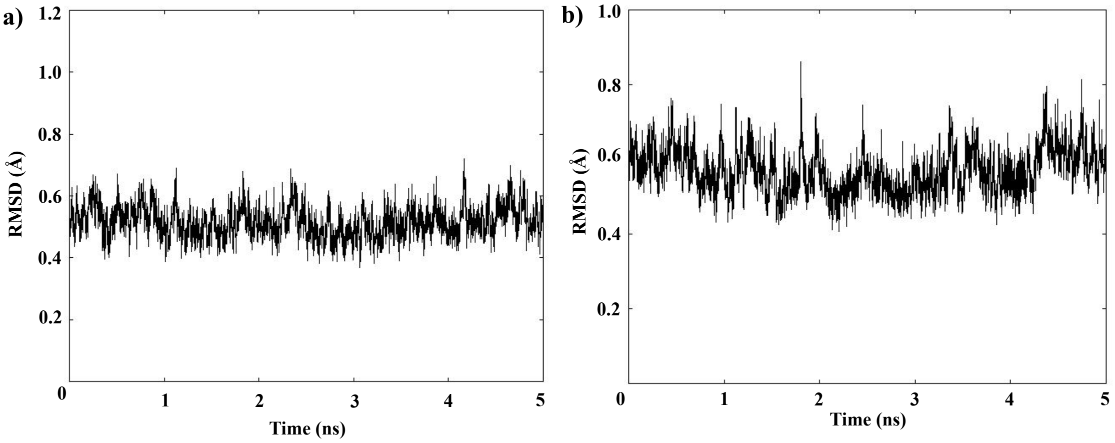

Figure S2. RMSD along the classical MD simulation for the backbone atoms of the cathepsin L cysteine protease. Simulations performed on the non-covalent reactant state corresponding to the **E·I** reactant complex. a) **11a**. b) **11b**.

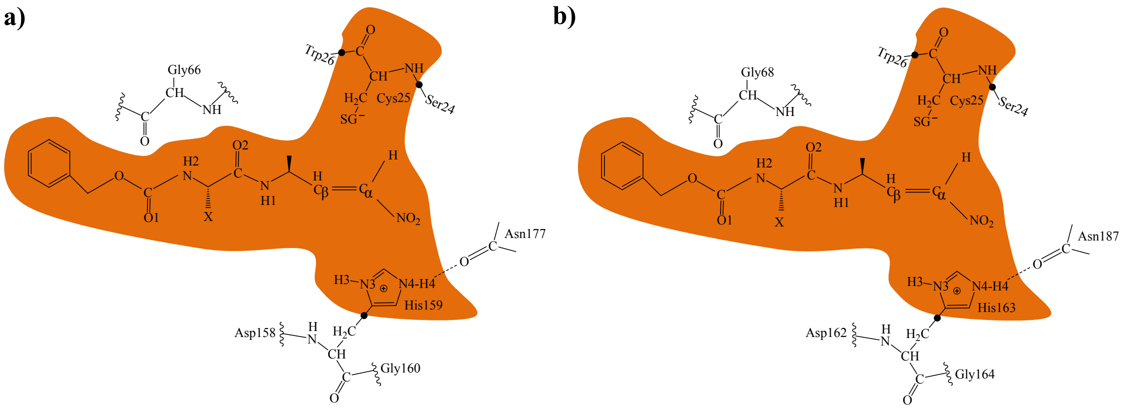

Figure S3. Details of the atoms of the active site treated quantum mechanically (orange region). Black dots represent the hydrogen link atoms. X corresponds to Trp (**11a**) and 4-NO_2-Phe (**11b**). Residue numbers of cruzain (a) and cathepsin L cysteine proteases (b).

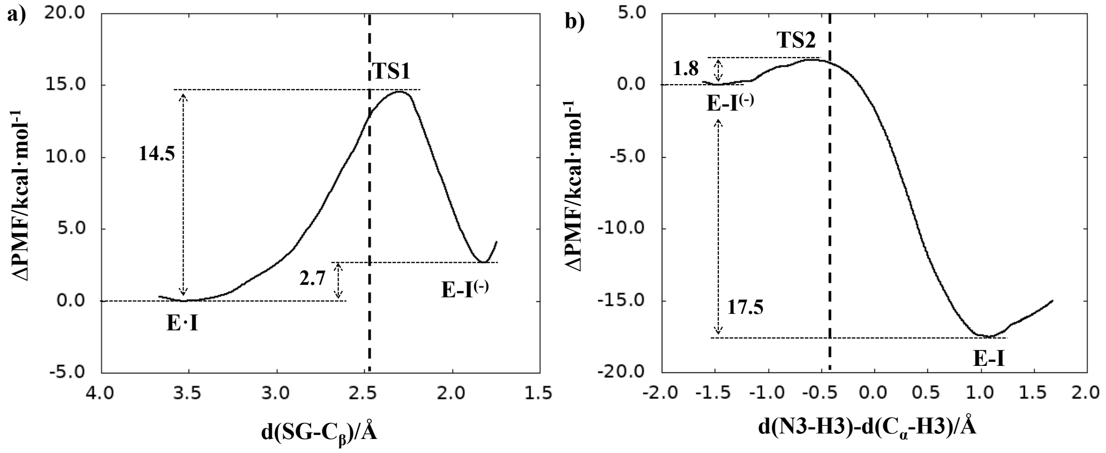

Figure S4. M06-2X/6-31+G(d,p):AM1d/MM FES for the inhibition mechanism of cruzain by the inhibitor **11a**. a) Attack of sulfur on C_β. b) Protonation of the INT-C_β intermediate. The position of the optimized TSs at M06-2X/6-31+G(d,p)/MM level are indicated as dashed vertical lines.

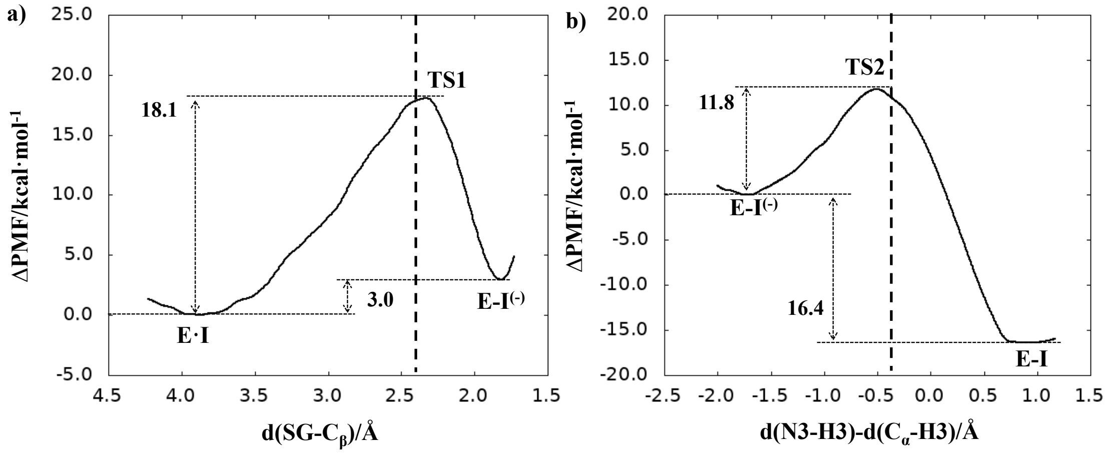

Figure S5. M06-2X/6-31+G(d,p):AM1d/MM FES for the inhibition mechanism of cruzain by the inhibitor **11b**. a) Attack of sulfur on C_β. b) Protonation of the INT-C_β intermediate. The position of the optimized TSs at M06-2X/6-31+G(d,p)/MM level are indicated as dashed vertical lines.

Table S3. Cartesian coordinates (in Å) of QM atoms TSs corresponding to the inhibition mechanism of cruzain by the inhibitor **11a**, optimized at M06-2X/6-31+G(d,p)/MM level.

TS1 (νi = 263.3i cm^{-1})				TS2 (νi = 491.7i cm^{-1})			
Atoms	x	y	z	Atoms	x	y	z
N	6.261603347405	10.252299735089	2.932125045037	N	6.360690841515	10.446739106499	2.859739536166
H	5.730586501494	11.116790967867	2.935421531390	H	5.885584318113	11.342279404147	2.902390062970
C	6.265876845423	9.532393128496	4.196725318236	C	6.426862676901	9.718714047222	4.124439749373
H	7.262851212823	9.493532054379	4.653378510594	H	7.433698120946	9.685403201885	4.554149756470
C	5.319161004335	10.114609699857	5.253063547266	C	5.487250367616	10.243803876232	5.211116848427
H	5.458519729081	9.523687844778	6.165917804475	H	5.530199540194	9.563206336811	6.068197802843
H	5.652199525500	11.132777122405	5.450695423379	H	5.845462767116	11.220967131613	5.529097631763
S	3.567923038616	10.227737492554	4.752112731104	S	3.765878458171	10.458865869507	4.628842897013
C	5.876991705797	8.119821890617	3.792458208959	C	5.988690415878	8.326641463267	3.736233896551
O	6.090093250511	7.081600182747	4.372434937214	O	6.116544539194	7.286868022111	4.333696514414
C	2.865699391273	8.787905493865	9.180744354020	C	2.879461142896	8.919089134809	9.100928569794

N	3.932671873564	9.241194956871	8.437570033556	N	3.880591804543	9.622969657808	8.461919023553
H	3.856850829384	9.758421086599	7.561058205347	H	3.715293772590	10.517971179280	7.701958804145
C	5.064296378641	9.008455184319	9.087511938636	C	5.027244423589	9.313699463073	9.047967906960
H	6.058775225290	9.288584600817	8.772279267600	H	6.000585726435	9.698035831073	8.789848555901
N	4.755595960921	8.402146353904	10.228786648787	N	4.801301142013	8.445585572019	10.036971325335
H	5.480406718106	8.125008968887	10.908526759992	H	5.540533183261	8.095446805548	10.663535346580
C	3.389467046477	8.253036653416	10.321414788933	C	3.456693627092	8.182094386155	10.095328035444
H	2.907596550700	7.787815555899	11.167256186778	H	3.027993096208	7.508525761392	10.819282647713
C	-1.365210189103	11.549153048445	-2.897387275625	C	-1.440379847983	11.489923065421	-2.887118819844
C	-1.734007273435	10.326433555261	-3.468878996956	C	-1.791292914315	10.274604095887	-3.486932161313
C	-1.253512208613	9.968101318444	-4.725253155416	C	-1.307754400728	9.952839990480	-4.752944903283
C	-0.426803717378	10.843522759218	-5.431873314027	C	-0.494266681198	10.856088449543	-5.441203393639
C	-0.068788778289	12.068272627597	-4.873442077119	C	-0.158128058666	12.076079384339	-4.856843267380
C	-0.529625550540	12.414438862875	-3.602929380820	C	-0.623949552569	12.386117272512	-3.578839312378
H	-2.388138655823	9.649778929411	-2.923971669566	H	-2.438263102035	9.575911183072	-2.960931619792
H	-1.509758463015	9.006032803927	-5.154075316090	H	-1.556446641909	8.998899515963	-5.205418050706
H	-0.047770149090	10.555452789122	-6.405416776819	H	-0.111659366131	10.597457944453	-6.421942715624
H	0.576935050161	12.744329239075	-5.423800749449	H	0.472303289963	12.776980768851	-5.394869393497
H	-0.229518593380	13.357513173682	-3.152102439089	H	-0.346371042047	13.328732188879	-3.112316489430
C	-1.820211202822	11.884690663740	-1.508494126885	C	-1.897238549573	11.795868177805	-1.490677857840
H	-2.896262966943	11.741806461753	-1.372274944156	H	-2.964458291135	11.602909319777	-1.347688417840
H	-1.562951546464	12.909435532538	-1.231717427903	H	-1.688238539758	12.830844978403	-1.213473166326
O	-1.128821675478	10.973799065615	-0.618410958789	O	-1.158348465459	10.917970887327	-0.602052593449
C	-1.105227004313	11.314325709995	0.681144368224	C	-1.069873329421	11.290837846177	0.687158540877
O	-1.709301880679	12.260249877507	1.156682148243	O	-1.652725247572	12.243519884885	1.174091197693
N	-0.318894718686	10.460912774245	1.381290152312	N	-0.230517610610	10.464489537155	1.363718660198
H	0.047355637950	9.632765067995	0.917848509195	H	0.072857876024	9.607734887695	0.904132611155
C	-0.148242125924	10.616690615804	2.801903272374	C	-0.045134411512	10.597864602696	2.788685834884
H	-1.134088056851	10.682781934685	3.278318404600	H	-1.025193248557	10.681647310150	3.276181452878
C	0.617338067448	11.914160163210	3.119157354228	C	0.762761614358	11.872537785931	3.125600003081
O	0.949401475180	12.715826685721	2.254055292077	O	1.066752424697	12.695861683682	2.274405007351
C	0.686279208154	9.450480053785	3.357284916857	C	0.744875518397	9.385000872215	3.318186204501
H	0.669219387977	9.516872867019	4.446889779559	H	0.721902783606	9.431220467997	4.409317383243
H	1.728607343418	9.628013080602	3.076185384197	H	1.794890484958	9.527719900438	3.042699702909
C	0.315183878115	8.073422081777	2.921214108715	C	0.319437323994	8.030666556177	2.852896532662
C	1.229266536624	7.084662655768	2.673403415357	C	1.190889860032	7.016985433248	2.554122242018
H	2.305292395892	7.135391088369	2.746966739998	H	2.268758692735	7.023693576580	2.572569668044
N	0.596984140733	5.917656636589	2.341494202931	N	0.512278252677	5.875281789039	2.231474897626
H	1.071145025869	5.050568628638	2.104530022708	H	0.959303499059	4.996767656708	1.987930904837
C	-0.761381665569	6.127866212765	2.371103603220	C	-0.836531586474	6.123549785557	2.319655278911
C	-0.979253745752	7.478125715858	2.742326965198	C	-0.998481074627	7.476332183905	2.710466412807
C	-1.815473235780	5.242748436454	2.122749984497	C	-1.923686375978	5.271040222147	2.105567142024
H	-1.630303473184	4.211542114585	1.837463450491	H	-1.776941693971	4.238301036385	1.805875056648
C	-3.102106970078	5.738190835674	2.239168430436	C	-3.190606314885	5.802112297045	2.268954570039
H	-3.945009362974	5.092412695168	2.042931782727	H	-4.057915651774	5.179595706912	2.102006301240
C	-3.345052717602	7.080342876742	2.597250528284	C	-3.378603856806	7.150025214862	2.637338101057
H	-4.369784946950	7.428341823426	2.665683843228	H	-4.388289880973	7.530095508539	2.740450495836
C	-2.299887782687	7.950276966665	2.855077639205	C	-2.299793055580	7.986819813157	2.865371139264
H	-2.504484411498	8.978848919002	3.136032974633	H	-2.465111629590	9.019759246504	3.155121971017
N	0.996403947149	12.068865677256	4.409073998266	N	1.216713619522	11.965382346107	4.405314114074
H	0.584741531187	11.536086126473	5.175095131917	H	0.735234700539	11.497473986399	5.174705316593
C	2.022850972925	13.023170885511	4.751316398922	C	2.325609820003	12.841412894427	4.752426252541
H	2.502543005201	13.272841684362	3.802902999903	H	2.747117078884	13.147066826278	3.789886113516
C	1.442461798152	14.302734898152	5.373797410803	C	1.896830480669	14.085590018436	5.525365699748
H	0.796691410288	14.793952172368	4.641953454281	H	1.237612553588	14.694774154571	4.903783107475
H	2.231618911941	14.996937200282	5.675787474326	H	2.766080965387	14.686798070715	5.809848411561
H	0.846676446495	14.058490408077	6.257811889166	H	1.354480778342	13.822905547702	6.436948879763
C	3.111240251507	12.492881416673	5.675129779446	C	3.432639119541	12.057982173004	5.502857012875
H	4.071326533256	12.922891828782	5.410987253226	H	4.341883507815	12.643642230680	5.368020740466
C	3.034068895788	12.271921434805	7.036118543668	C	3.278883161289	11.847121722785	6.982876048410
H	2.290855345432	11.709262404327	7.576962873434	H	2.270586436684	11.759131741456	7.374627644401
N	4.180476376712	12.540560441494	7.781979543438	N	3.989200596710	12.749240167725	7.795677249922
O	4.985238973644	13.392592614927	7.397640059456	O	4.927605575077	13.421973999773	7.323252848181
O	4.363809795121	11.905906749092	8.838586624608	O	3.725226089978	12.793577737947	9.000135759480

Table S4. Cartesian coordinates (in Å) of QM atoms TSs corresponding to the inhibition mechanism of cruzain by the inhibitor **11b**, optimized at M06-2X/6-31+G(d,p)/MM level.

TS1 (νi = 156.5i cm^{-1})				TS2 (νi = 646.9i cm^{-1})			
Atoms	x	y	z	Atoms	x	y	z
N	1.254704272443	8.821014084993	-4.823596706389	N	0.760538265767	10.353704503338	-5.945247477937
H	0.948032078541	9.789312886485	-4.948187386595	H	0.421037443859	11.287609601585	-5.743966496047
C	0.728031718404	8.264603847741	-3.592817032895	C	1.448440961713	9.738638680096	-4.815561162701
H	1.439141594892	8.266259705310	-2.773959655388	H	2.539980080543	9.741390887950	-4.905942899017
C	-0.597423270998	8.940793155260	-3.221942586723	C	1.037246521943	10.355962415300	-3.462682716565
H	-0.566091394044	9.972489710554	-3.582596121538	H	1.538356825884	9.793189097742	-2.668763566052
H	-1.427217252558	8.473331150730	-3.768188620005	H	1.340972113082	11.400818578944	-3.399065727874
S	-0.906087984241	8.951306776248	-1.444159899991	S	-0.789673052604	10.206836453100	-3.280712882275
C	0.387770919718	6.864839594876	-3.980236550428	C	0.911477395070	8.322283229758	-4.783484304652
O	0.588756274670	5.824954124727	-3.397062174777	O	1.339914864114	7.374729794121	-4.171369426951
C	-2.617059455519	5.232549745391	1.894341343719	C	-1.182265089657	5.706197010994	0.618072931370
N	-1.450111198595	5.537504330861	1.222531953055	N	-0.631609124369	6.624745482768	-0.257685882755
H	-1.354973900725	5.859733111859	0.234608786331	H	-0.961599179847	7.739518284761	-0.535674349179
C	-0.404841176514	5.127657807534	1.923628622472	C	0.656220171064	6.347101420616	-0.370493488135
H	0.627730074891	5.233589873673	1.626404305272	H	1.382847446500	6.912382062813	-0.938582691518
N	-0.857033513244	4.581813237301	3.050087215126	N	0.945636847001	5.273691133371	0.379315022416
H	-0.241277679723	4.155805410002	3.752417702810	H	1.865752917987	4.866099387812	0.478050681851
C	-2.234087552636	4.630176830925	3.057985800064	C	-0.195086523464	4.849034654117	1.016420683093
H	-2.817468100559	4.246593068618	3.879244883161	H	-0.202866541126	3.988240502758	1.667539706444
C	-9.857502731844	10.247131647540	-5.774379281304	C	-10.494868250367	12.073217691860	-3.904642795075
C	-10.002936225655	9.281498181651	-4.770385901377	C	-10.817473882415	10.796772114909	-3.429736991684
C	-10.753570652476	8.131806763983	-5.007655733953	C	-12.038555651497	10.215537012112	-3.760423583917
C	-11.372895888760	7.940321446175	-6.242339205642	C	-12.959037069628	10.912126487972	-4.546248011796
C	-11.224501796665	8.892309003686	-7.251779440399	C	-12.643740642416	12.187871628926	-5.014709559895
C	-10.467315146783	10.040978914338	-7.017867429714	C	-11.409581286576	12.762281451174	-4.702927324343
H	-9.522074523733	9.424380180011	-3.808421511322	H	-10.108286870896	10.260287636436	-2.802218299004
H	-10.849479850685	7.387162054110	-4.223971120575	H	-12.279693353751	9.216342583744	-3.411490827144
H	-11.969505780603	7.050727133341	-6.419991864347	H	-13.917397945219	10.462011807635	-4.786877822618
H	-11.694536780864	8.742937499557	-8.218969249729	H	-13.353896345258	12.726293749072	-5.634867052318
H	-10.344562432463	10.776462878468	-7.809969117551	H	-11.151606825029	13.746337680186	-5.088473630705
C	-9.064880224043	11.506771763422	-5.531660017286	C	-9.159632901610	12.685804355975	-3.578594463689
H	-9.246167312559	11.910118752607	-4.532825582077	H	-8.957335316151	12.662436736150	-2.505902627123
H	-9.304748450649	12.260084702041	-6.284403512529	H	-9.099145823243	13.712042877609	-3.946319796076
O	-7.640051718042	11.299663432393	-5.687892318209	O	-8.101598442515	11.982672999300	-4.261458268917
C	-6.980219001743	10.843522686191	-4.612680574271	C	-7.353889018874	11.120649190582	-3.568490984990
O	-7.482172916366	10.634295356597	-3.520083538012	O	-7.530802589934	10.838564886893	-2.381032700097
N	-5.671700858099	10.631926609298	-4.901766171812	N	-6.401008700959	10.559690558745	-4.346425829874
H	-5.304239705978	10.969133840024	-5.782968914715	H	-6.202468650756	11.051313734363	-5.226373444930
C	-4.764174964837	10.085636802524	-3.926936896044	C	-5.344673078126	9.801073221377	-3.709127077633
H	-5.345480959949	9.839110514786	-3.034446773259	H	-5.812212811071	9.113581708674	-2.994356879293
C	-3.669146225874	11.107390414448	-3.611307510461	C	-4.401856550814	10.734086693672	-2.916803434313
O	-3.282034352379	11.889499144980	-4.485305009756	O	-4.505226804169	11.954267475877	-3.012645706747
C	-4.056112546466	8.815659170601	-4.456211378703	C	-4.558815889851	8.993232528878	-4.769675730781
H	-3.472849884622	9.106010556903	-5.341965656277	H	-4.726917549932	9.452982605987	-5.749706282585
H	-3.359015742912	8.497366931294	-3.676060363092	H	-3.489263959377	9.088289307735	-4.554578214741
C	-4.969417618640	7.658305735768	-4.775234514404	C	-4.912899999146	7.525056784646	-4.815134610302
C	-5.861962892997	7.694958246068	-5.855744444204	C	-5.897892189268	7.025943754715	-5.673405208403
H	-5.913025993649	8.573107529101	-6.491950252141	H	-6.468491932880	7.706924466983	-6.299442090485
C	-6.712922879620	6.626627450737	-6.110261008253	C	-6.147668456319	5.656991507312	-5.754470684603
H	-7.426045281334	6.662352167983	-6.923948824350	H	-6.879529715978	5.247931344142	-6.441860869663
C	-6.633152360487	5.508319999944	-5.290435291588	C	-5.382849086094	4.804319801415	-4.970226874629
N	-7.540754415538	4.382597352887	-5.520035311940	N	-5.570493146621	3.349824979146	-5.094745369186
O	-8.450852886809	4.533464170745	-6.328693314133	O	-6.547433501801	2.953648236646	-5.698701169172
O	-7.369152098189	3.365092149239	-4.888339411887	O	-4.729921197627	2.633949656185	-4.571486104950
C	-5.744983588260	5.421763466796	-4.227982634758	C	-4.417803854704	5.263712622603	-4.083625007928
H	-5.726481421815	4.534867904494	-3.605744897975	H	-3.831986110539	4.551959325013	-3.514423270995
C	-4.919178989289	6.509882276081	-3.977728868491	C	-4.205813442461	6.632335872644	-4.000242578049
H	-4.240849320166	6.483113550560	-3.129846087262	H	-3.450766664051	7.017735932635	-3.317542182515
N	-3.101416774965	11.040858730946	-2.394648663401	N	-3.457021599358	10.115186954543	-2.163737072633
H	-3.419871562817	10.351799982514	-1.721587017833	H	-3.676038066883	9.169907689385	-1.828175976745
C	-1.895415392399	11.821721401030	-2.145454818380	C	-2.547106434056	10.913465895419	-1.336644120007
H	-1.355552934599	11.781630845152	-3.095094380522	H	-2.606286806302	11.922540909984	-1.751259350254
C	-2.220151224863	13.292131348926	-1.848989760017	C	-3.069067235007	10.938808053750	0.097688748559
H	-1.313951931551	13.854004598136	-1.608454614527	H	-4.122067504595	11.224885551091	0.082750355914
H	-2.895176863020	13.366099087375	-0.994006249579	H	-2.516043270207	11.656347199753	0.700041274377
H	-2.688880729822	13.737423967006	-2.727219962752	H	-3.003904969998	9.950168450364	0.565959942224
C	-0.980326765008	11.296506351005	-1.051019885238	C	-1.057151060470	10.483673625855	-1.482264246420
H	0.077294207666	11.396096258331	-1.282603860236	H	-0.464588478148	11.369319303276	-1.215352551043
C	-1.280700874806	11.405332587708	0.294122027598	C	-0.590027726252	9.285362202244	-0.662963665223

H	-2.270383566909	11.337523874156	0.726697084718	H	0.348640837449	8.877293407667	-1.050357101775
N	-0.249579696770	11.213863570239	1.196926251286	N	-0.318155361055	9.592859903987	0.708414800038
O	-0.540505375068	10.710169378595	2.303771152638	O	-0.054151686435	8.655188423742	1.488845367335
O	0.927224354085	11.497545946558	0.903478890456	O	-0.393111075734	10.750007448354	1.136574219761

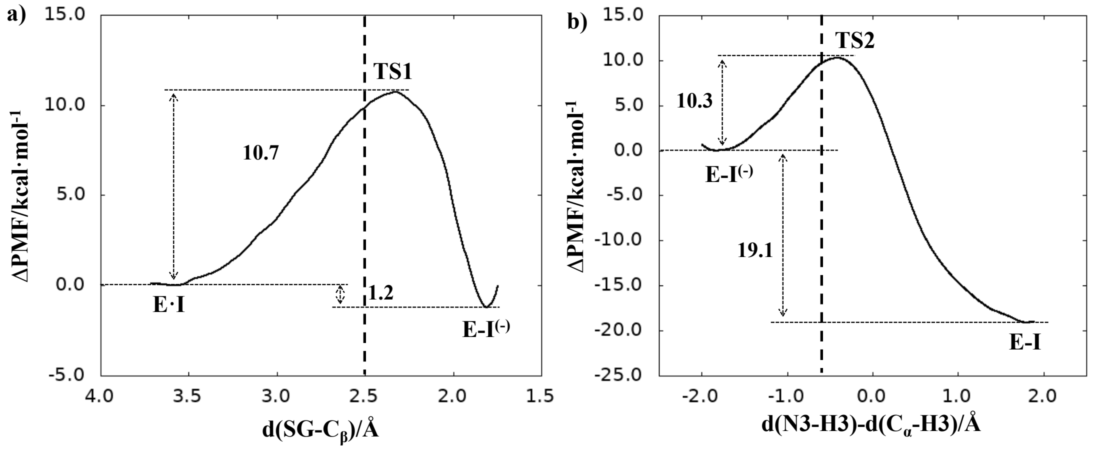

Figure S6. M06-2X/6-31+G(d,p):AM1d/MM FES for the inhibition mechanism of cathepsin L by the inhibitor **11a**. a) Attack of sulfur on $C_β$. b) Protonation of the INT-$C_β$ intermediate. The position of the optimized TSs at M06-2X/6-31+G(d,p)/MM level are indicated as dashed vertical lines.

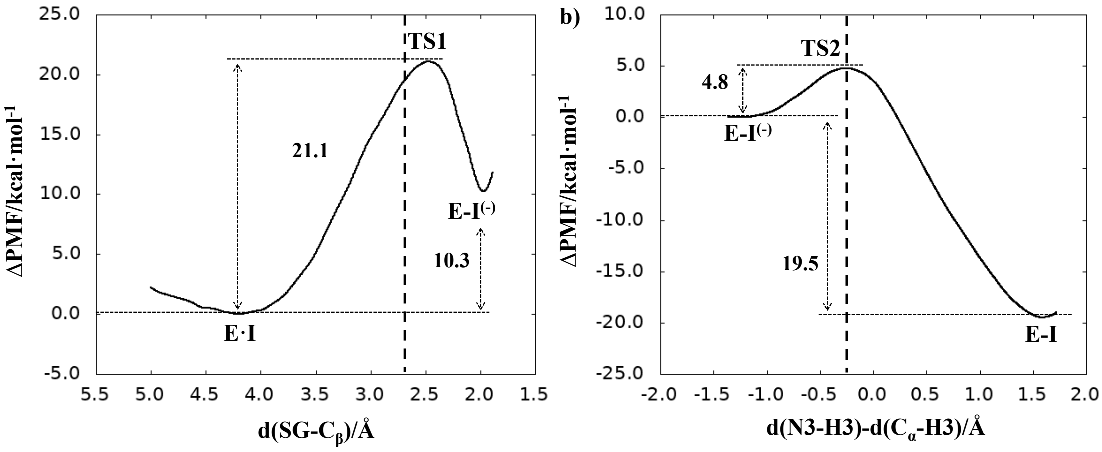

Figure S7. M06-2X/6-31+G(d,p):AM1d/MM FES for the inhibition mechanism of cathepsin L by the inhibitor **11b**. a) Attack of sulfur on $C_β$. b) Protonation of the INT-$C_β$ intermediate. The position of the optimized TSs at M06-2X/6-31+G(d,p)/MM level are indicated as dashed vertical lines.

Table S5. Cartesian coordinates (in Å) of QM atoms of the TSs corresponding to the inhibition mechanism of cathepsin L by the inhibitor **11a**, optimized at M06-2X/6-31+G(d,p)/MM level.

TS1 (νi = 56.7i cm^{-1})				TS2 (νi = 718.7i cm^{-1})			
Atoms	x	y	z	Atoms	x	y	z
N	11.572091348438	0.894380579188	1.027798015878	N	12.824815856727	2.814363278097	1.808404788306
H	12.255443094799	1.033987789362	1.752518622745	H	13.700208619889	3.333711335833	1.894332220514
C	11.506727817392	-0.379345576595	0.363851932729	C	12.808917826552	1.989227870928	0.598748479699
H	11.199110807154	-0.242533106331	-0.674642808560	H	12.389333601830	2.495640014746	-0.277921688508

C	12.861343016429	-1.112205545318	0.425485653300	C	14.215671407819	1.434948836312	0.261439267960
H	13.522558313339	-0.726630042931	-0.349775616728	H	14.206758308098	1.033196717128	-0.751067187066
H	13.341958324831	-0.919757599387	1.390170360884	H	14.957912938788	2.233549362025	0.297245805893
S	12.712348222187	-2.950855132847	0.293740102218	S	14.730441783525	0.087725714818	1.423053203668
C	10.402869312434	-1.176638553537	1.037946511890	C	11.983842195850	0.788693757852	1.008954051093
O	9.226051014132	-0.921858197660	0.898943405649	O	11.408096705402	-0.021336995758	0.323217533582
C	12.023290119068	-3.426271378041	-3.615443851143	C	12.693185564373	-2.667099980806	-3.348899831550
N	12.371345668762	-2.198680172701	-3.089382334263	N	13.353518557979	-1.704704082767	-2.567358109958
H	13.351686660414	-1.950486382478	-2.801202165732	H	14.378356134454	-1.638308334664	-1.975991808316
C	11.322776761762	-1.388902127784	-3.112551048051	C	12.576092696016	-0.623323479025	-2.549186291816
H	11.305479924240	-0.360511075483	-2.780454084771	H	12.787520801717	0.290754661998	-2.016037263053
N	10.299587363482	-2.062251995801	-3.644341147037	N	11.473318541431	-0.840369865636	-3.269977721725
H	9.363111360925	-1.673540567392	-3.818568673141	H	10.741246630838	-0.157811919757	-3.486212817465
C	10.709938214356	-3.331455847274	-3.973325613723	C	11.520890550895	-2.111286013953	-3.778004932049
H	10.034056201395	-4.048435661008	-4.407546605238	H	10.730847734786	-2.499213458063	-4.404224005102
C	18.321057690053	-3.215945619172	6.203675086245	C	18.619474808741	-2.363322381842	6.425633938884
C	18.568722668258	-1.839301496408	6.216626235263	C	18.996682794048	-1.056885161513	6.754827502103
C	18.301417375438	-1.081296384122	7.352637206655	C	18.738808363284	-0.551172025667	8.028311957751
C	17.774518954163	-1.692219100042	8.489693041074	C	18.100916368221	-1.345697193150	8.978059802333
C	17.539363622211	-3.065501428857	8.486347168287	C	17.734397427736	-2.651616552364	8.655633070133
C	17.814999681268	-3.830607818545	7.350181879347	C	17.993248185921	-3.162400889955	7.385992237613
H	18.965635837755	-1.357745122771	5.325599587499	H	19.485352558538	-0.431900840532	6.012617338163
H	18.475342689042	-0.010087750313	7.342268028323	H	19.018643090571	0.469047369220	8.268508901492
H	17.526696978452	-1.094759861687	9.359682275220	H	17.878125840579	-0.944266760546	9.960141130199
H	17.122045314301	-3.541736858170	9.368337411854	H	17.246639443892	-3.277754629014	9.394906208613
H	17.622152935426	-4.898627318652	7.358916180483	H	17.701723819490	-4.176500015651	7.135776192923
C	18.644225049194	-3.962247755611	4.933743155944	C	18.950171112175	-2.891205866342	5.053499009596
H	19.714799827585	-4.180110585289	4.857088908628	H	19.953715421758	-3.329640473960	5.019872327729
H	18.342365620031	-3.368622940360	4.066921476896	H	18.904037256405	-2.096538007756	4.303624210915
O	17.931767520380	-5.213805851156	4.903709482486	O	18.005563101574	-3.922229745799	4.719555399188
C	17.864503417833	-5.847000878487	3.735376306603	C	18.052452556157	-4.390393370101	3.473103449371
O	18.394854987061	-5.468863359238	2.691200692908	O	18.874522610784	-4.013472749663	2.635151446991
N	17.110441741544	-6.976169468398	3.839862023353	N	17.087113085335	-5.311681569861	3.253563708288
H	16.765537072903	-7.229744463195	4.758275680687	H	16.387297036701	-5.405159016084	3.981866155723
C	16.749301450381	-7.762245964873	2.682987405696	C	16.748452322981	-5.797470268276	1.926881716550
H	17.551299807220	-7.662226880886	1.947682227167	H	17.650205650487	-5.693044602479	1.312272341213
C	15.506160393809	-7.252547850661	1.944086240609	C	15.656696247533	-4.940415370806	1.250555147921
O	15.290658714612	-7.630583859114	0.794774652581	O	14.754716418802	-5.477586377123	0.607667850827
C	16.582206239958	-9.250286512900	3.026218044033	C	16.348726187362	-7.278295324073	1.940475918889
H	17.574719560103	-9.651869310745	3.248279545511	H	17.246593392869	-7.854707622535	2.184901528832
H	16.245062981809	-9.726407947067	2.102764490391	H	16.068331735274	-7.531216513321	0.914778862997
C	15.660851575833	-9.546336355573	4.181241131476	C	15.250363928413	-7.679790364342	2.890322257017
C	14.297836195701	-9.378181018103	4.276519173712	C	13.978693421987	-7.173452628736	3.023831260841
H	13.600037110972	-8.974186215426	3.553641087192	H	13.503461407437	-6.360532855778	2.492780880380
N	13.851409435829	-9.803518803065	5.510016354246	N	13.282126693342	-7.884376700447	3.977062678574
H	12.922216365154	-9.648949449404	5.884151189989	H	12.336136548098	-7.667899539666	4.266590071377
C	14.927246478973	-10.230713898579	6.255012081376	C	14.085488604762	-8.875622026575	4.484803869783
C	16.083823195772	-10.082646338300	5.452278666432	C	15.335337297782	-8.780245338057	3.818068217133
C	14.983584186825	-10.691137085094	7.576326680481	C	13.848065364867	-9.824292400762	5.488409707904
H	14.089737132890	-10.766479847985	8.185775202259	H	12.901140495145	-9.861841587432	6.013045145965
C	16.231334295782	-11.008757573666	8.087484793330	C	14.874698318957	-10.702324069489	5.800024431154
H	16.312636336325	-11.352820291575	9.113587123711	H	14.722702086980	-11.439240384776	6.583360494342
C	17.401571827981	-10.870188861513	7.306362911032	C	16.116273670313	-10.641982263993	5.131361492795
H	18.364211955093	-11.108665946588	7.749362808556	H	16.895368328601	-11.349227943186	5.400688015041
C	17.339522537343	-10.411926874931	5.998774252101	C	16.353774875532	-9.691166707948	4.149908429711
H	18.248424611576	-10.279435351989	5.416143425835	H	17.316757065837	-9.649172467494	3.649791825780
N	14.687989203937	-6.413360692060	2.617585960302	N	15.772358742709	-3.605375577978	1.402134177613
H	14.994266487877	-6.081776607794	3.526665274434	H	16.679582327568	-3.239785653796	1.673949048233
C	13.769112835448	-5.539178671889	1.877835935416	C	14.878405394523	-2.712222059721	0.652323465586
H	13.758151633395	-5.928134844884	0.857065038014	H	14.832946650703	-3.132216083945	-0.354464852896
C	12.377517775502	-5.619502563456	2.479199987735	C	13.491818069600	-2.768698309175	1.273610660945
H	12.362069203229	-5.243105641626	3.505915482275	H	12.814362142800	-2.055471803092	0.794985450614
H	12.052139354705	-6.663153482358	2.499162250527	H	13.572386938020	-2.544722100842	2.341041809420
H	11.672614848501	-5.051846417180	1.872001719391	H	13.087703694841	-3.778115387982	1.168440150868
C	14.433041404419	-4.172769272138	1.892359620941	C	15.529433516586	-1.297719027617	0.471090593083
H	14.198013401722	-3.513045816136	2.724326583408	H	16.506056446210	-1.326364363213	0.964703257408
C	15.613046993805	-3.993620670116	1.237123804680	C	15.776893509259	-1.057305248292	-1.018701405122
H	15.963319605291	-4.578201611719	0.398373313321	H	15.846361378155	-0.022895416337	-1.352791922151
N	16.360262469888	-2.846855931272	1.499308618690	N	17.029108699458	-1.665273137759	-1.375766652140
O	17.295452293377	-2.557839717403	0.724707718881	O	17.556985375941	-1.398321705352	-2.474372797202
O	16.092554860296	-2.135235487506	2.465906392858	O	17.566272622665	-2.470001541575	-0.610933262232

Table S6. Cartesian coordinates (in Å) of QM atoms of the TSs corresponding to the inhibition mechanism of cathepsin L by the inhibitor **11b**, optimized at M06-2X/6-31+G(d,p)/MM level.

TS1 (vi = 144.1i cm^{-1})				TS2 (vi = 1465.6i cm^{-1})			
Atoms	x	y	z	Atoms	x	y	z
N	5.547981535609	4.770926439933	2.246638041851	N	5.449372662900	5.118600951034	2.449492520249
H	6.256412026325	5.441681625930	1.955744906829	H	6.247008998429	5.744903625952	2.422590540429
C	5.011410242895	4.111370744803	1.070429657870	C	5.138449363672	4.560990196420	1.154445288889
H	4.312374264746	4.720072926203	0.479865945971	H	4.350518927488	5.098639228245	0.610842194235
C	6.121270081993	3.578544671871	0.114772820508	C	6.361564515092	4.390418316065	0.214632827999
H	6.656381158221	2.758549394056	0.611823716819	H	6.983711650711	3.557239452939	0.549026313392
H	5.586281614719	3.159957416791	-0.747796633285	H	5.976271786503	4.164754647301	-0.785335677298
S	7.352127495330	4.786861928800	-0.492448975581	S	7.429589384860	5.849106197536	0.138367757808
C	4.312135140502	2.828663364906	1.419748234343	C	4.681097564344	3.141806268065	1.356334644232
O	3.302012088003	2.442640428428	0.862327132308	O	3.766085671360	2.630891743582	0.736674974633
C	6.999332213523	3.424035560857	-4.738616953298	C	7.996837449443	3.230583321304	-3.639650516196
N	6.723033827610	4.473309453642	-3.882083089597	N	8.037967448991	4.368093134650	-2.854730925101
H	7.193584857058	4.695432109811	-2.986040056339	H	8.834300956492	4.530415074454	-1.904953633242
C	5.615272332603	5.081523036174	-4.288874694104	C	6.958505589502	5.083351624917	-3.132132416310
H	5.181799439230	5.959340619284	-3.840684187610	H	6.701910356178	6.030964250044	-2.681972548271
N	5.174181902894	4.456044748979	-5.375636134445	N	6.225362719836	4.442471376617	-4.056095229018
H	4.315901367129	4.701567772317	-5.869554440384	H	5.310153755917	4.740387258058	-4.410049814232
C	6.015596287173	3.417264841270	-5.680625804272	C	6.854672666591	3.272279876493	-4.388959643219
H	5.840965968344	2.764512357067	-6.518898658305	H	6.435445241752	2.585431716132	-5.104058926121
C	12.419317175265	0.706748588567	7.543334590545	C	12.051755809930	0.945646281372	7.579033435465
C	11.462622340316	-0.010967061635	8.271153347459	C	11.510779492145	-0.237784630798	8.092648719804
C	11.360579399677	0.157274480970	9.650852037511	C	11.195085737354	-0.341545216879	9.442272357672
C	12.233144104520	1.019799807478	10.315599857902	C	11.422522036620	0.738846239814	10.296541616318
C	13.189301631067	1.731421222495	9.595073250722	C	11.989841722421	1.908664468228	9.800036635256
C	13.272925426653	1.586341251402	8.210235059195	C	12.309022033620	2.010281888840	8.445117437995
H	10.785954318274	-0.687428121636	7.756503367189	H	11.313427446223	-1.075045442295	7.428102919864
H	10.598742109708	-0.375682044677	10.211755252746	H	10.769701420344	-1.261170327852	9.830049654997
H	12.166197031705	1.140642670099	11.390551586930	H	11.148190028198	0.670154844810	11.343442878789
H	13.867481544608	2.403245026948	10.111311117066	H	12.163414993246	2.751150594073	10.462151357380
H	14.003660204709	2.163459736397	7.649949999614	H	12.730594506435	2.937930635084	8.062546192503
C	12.568765571946	0.503932056113	6.057534468307	C	12.275312686109	1.073582142905	6.095733098446
H	13.114934795146	-0.419054303701	5.836378006686	H	12.996857228808	0.349178176801	5.705259763449
H	13.091876614202	1.340028768909	5.585011584572	H	12.593732710473	2.080162978864	5.813940962542
O	11.235625445057	0.389837500164	5.510583929860	O	10.991296133563	0.794972303730	5.470590233996
C	11.068127925023	0.208821197787	4.205986744828	C	10.850242437910	1.001561571980	4.159254351786
O	11.967356478159	0.309421288189	3.357534012301	O	11.693387813197	1.504296039946	3.430282143538
N	9.777419513876	-0.079107105569	3.925366930929	N	9.629578725821	0.577769630003	3.733074167867
H	9.124506188909	-0.079236819199	4.731183381212	H	8.979795675916	0.224120685512	4.445167892238
C	9.234772678223	-0.128765691852	2.577649883608	C	9.287423089701	0.669299795059	2.324860414102
H	10.093647068316	-0.178219036455	1.898708862044	H	10.229349640240	0.599475511976	1.774452792598
C	8.489138521432	1.204890174290	2.320229436898	C	8.663508814888	2.044220774056	2.046232765886
O	7.259107694039	1.288277100013	2.346781052075	O	7.434027400216	2.191467023356	2.102158275824
C	8.300535508896	-1.321546775666	2.376785863062	C	8.346127163621	-0.453879058228	1.881660112606
H	7.484721842704	-1.257557482661	3.100709054909	H	7.429733176145	-0.454636257425	2.472592349812
H	7.820937187254	-1.209966083166	1.396972792195	H	8.043087192682	-0.242970201633	0.849199321756
C	8.927485410588	-2.700549152927	2.447655861401	C	8.991467975169	-1.814155290214	1.939974956810
C	10.134052475295	-3.017295905013	1.806978749926	C	10.089018118317	-2.091485705727	1.114030613268
H	10.707039531117	-2.243474309393	1.305275096231	H	10.480180195212	-1.318496289619	0.458331849355
C	10.620885397142	-4.321171712158	1.784527812846	C	10.677541699082	-3.343853963113	1.116314490523
H	11.553580890206	-4.561348003241	1.288967163798	H	11.526631722430	-3.577827592858	0.486655138188
C	9.876893794829	-5.316762056971	2.412740167529	C	10.149601394015	-4.316147385806	1.963017124850
N	10.326326115856	-6.712492882847	2.316601458358	N	10.774724844611	-5.635135129338	1.954366902649
O	11.387518124526	-6.938200334235	1.730100417333	O	11.631662128005	-5.838529434962	1.093095855593
O	9.617764181080	-7.582272552396	2.777158834684	O	10.428036141862	-6.463185568027	2.769544853928
C	8.687796198526	-5.043348246807	3.078850159932	C	9.071156421828	-4.079024278148	2.803205742102
H	8.130326505928	-5.828107466448	3.573666767642	H	8.682143022434	-4.858898896859	3.447433847585
C	8.230442806032	-3.729669742283	3.089804893393	C	8.496209392925	-2.811451350132	2.782376929066
H	7.295479153375	-3.517425161919	3.592418492739	H	7.639434847468	-2.611023066555	3.416800359651
N	9.316579354365	2.252069584671	2.102138151215	N	9.522347946651	3.034530318260	1.776525681657
H	10.308108920711	2.069168471525	2.235464125905	H	10.506195683667	2.771487997400	1.771684594974
C	8.902080796387	3.639003475413	2.317368760402	C	9.212980514607	4.467504221577	1.861868436025

H	7.809919225747	3.665425382245	2.335979140439	H	8.250178296883	4.542207972601	2.376830666581
C	9.442654895277	4.103245697141	3.683021069702	C	10.304429873957	5.117418692205	2.708783302760
H	9.200271938627	5.147172043830	3.871731615868	H	10.059909376342	6.156995641729	2.924196353531
H	10.532405024595	4.000923307173	3.711694440383	H	11.262913659189	5.086394069449	2.178373782596
H	8.992817607484	3.497424879514	4.474397782649	H	10.420271335387	4.592129539725	3.657997810827
C	9.391132635797	4.592814347032	1.261800375689	C	9.118018426211	5.215153757908	0.480562660759
H	9.071141983031	5.617220532157	1.401514169357	H	9.652953477283	6.162143688570	0.598395888386
C	10.549667979751	4.395370827796	0.554958533771	C	9.729749058154	4.497878487926	-0.697218414789
H	11.084760186501	3.463184769563	0.433000726896	H	9.882864692555	3.435601346545	-0.558521870608
N	11.092352543279	5.459063332257	-0.146571675853	N	10.941386040097	5.018472962753	-1.190478309658
O	12.211479816948	5.326050965539	-0.699192304205	O	11.659976996926	4.264248460864	-1.876684986286
O	10.468721783251	6.534050153448	-0.231234833266	O	11.270088868776	6.198557344512	-0.999278890929

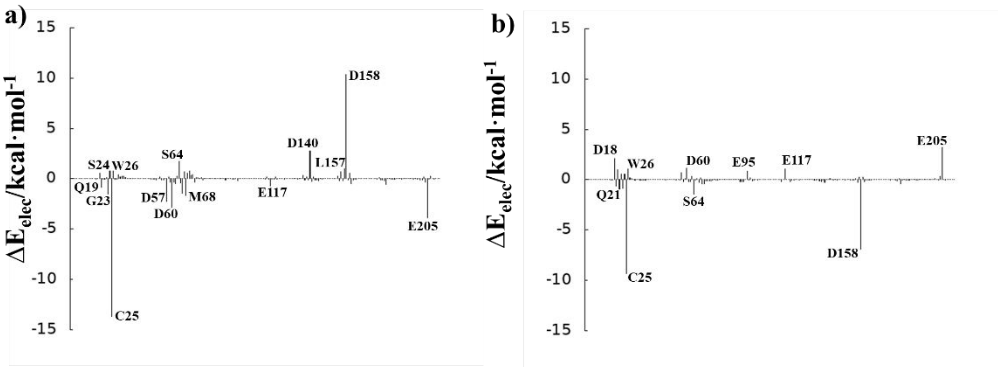

Figure S8. Averaged electrostatic interaction energies between residues of cruzain and the inhibitor **11a** (a) and the inhibitor **11b** (b) computed in the **E·I** reactant complex. Results obtained as an average over 10000 structures from the AM1d/MM MD simulations.

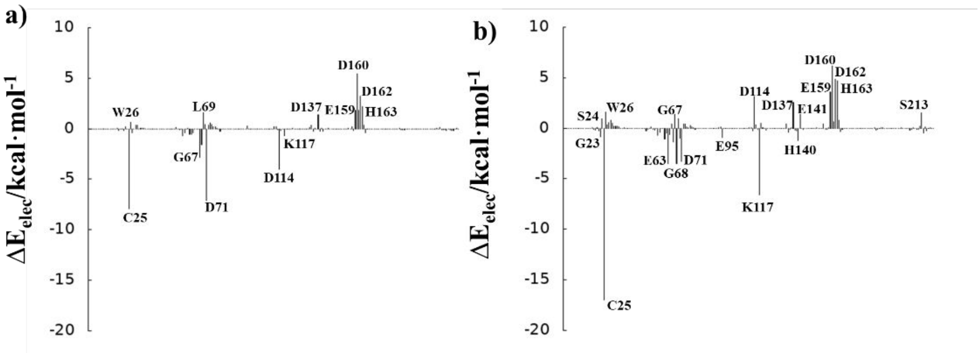

Figure S9. Averaged electrostatic interaction energies between residues of cathepsin L and the inhibitor **11a** (a) and the inhibitor **11b** (b) computed in the **E·I** reactant complex. Results obtained as an average over 10000 structures from the AM1d/MM MD simulations.

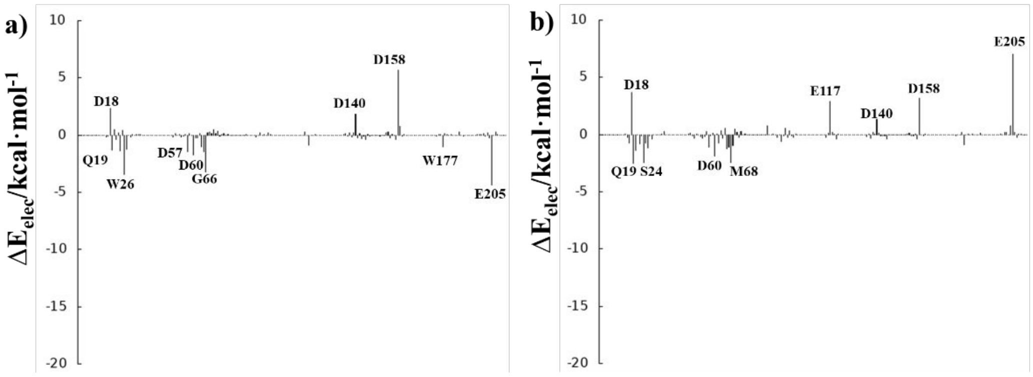

Figure S10. Averaged electrostatic interaction energies between residues of cruzain and the inhibitor **11a** (a) and the inhibitor **11b** (b) computed in the **E-I** reactant complex. Results obtained as an average over 10000 structures from the AM1d/MM MD simulations.

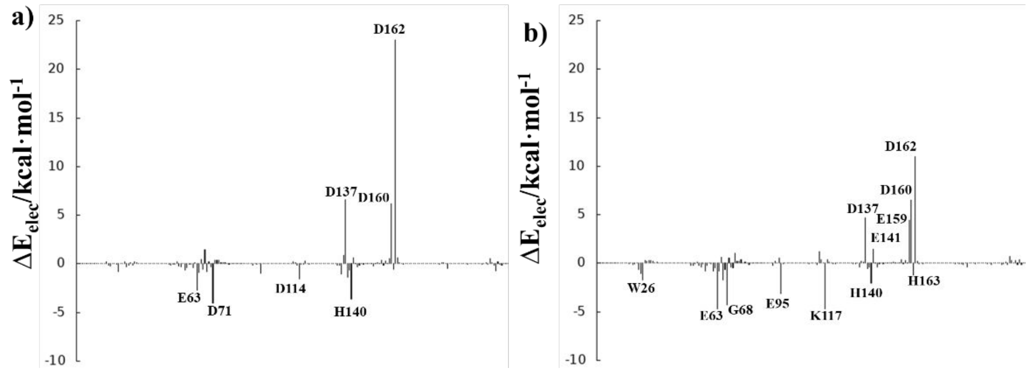

Figure S11. Averaged electrostatic interaction energies between residues of cathepsin L and the inhibitor **11a** (a) and the inhibitor **11b** (b) computed in the **E-I** reactant complex. Results obtained as an average over 10000 structures from the AM1d/MM MD simulations.

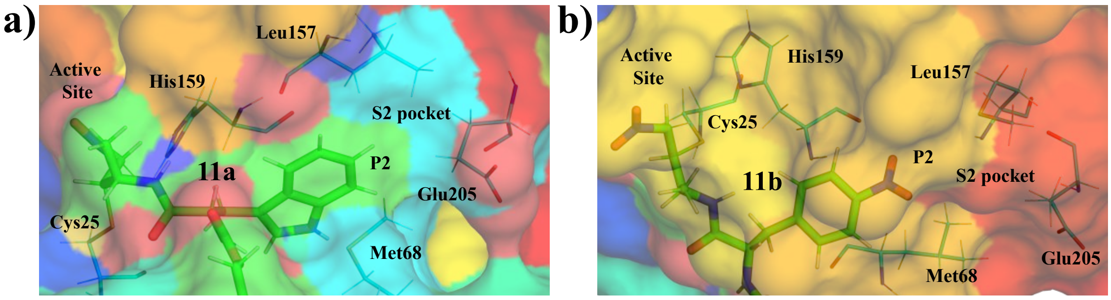

Figure S12. a) Detail of the structures of **E-I** covalent complex appearing along the inhibition process of cruzain by inhibitor **11a** (a) and inhibitor **11b** (b).

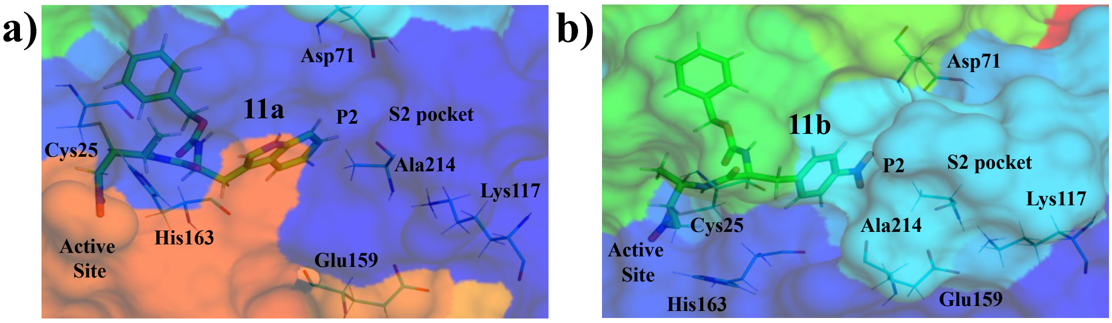

Figure S13. a) Detail of the structures of **E-I** covalent complex appearing along the inhibition process of cathepsin L by inhibitor **11a** (a) and inhibitor **11b** (b).

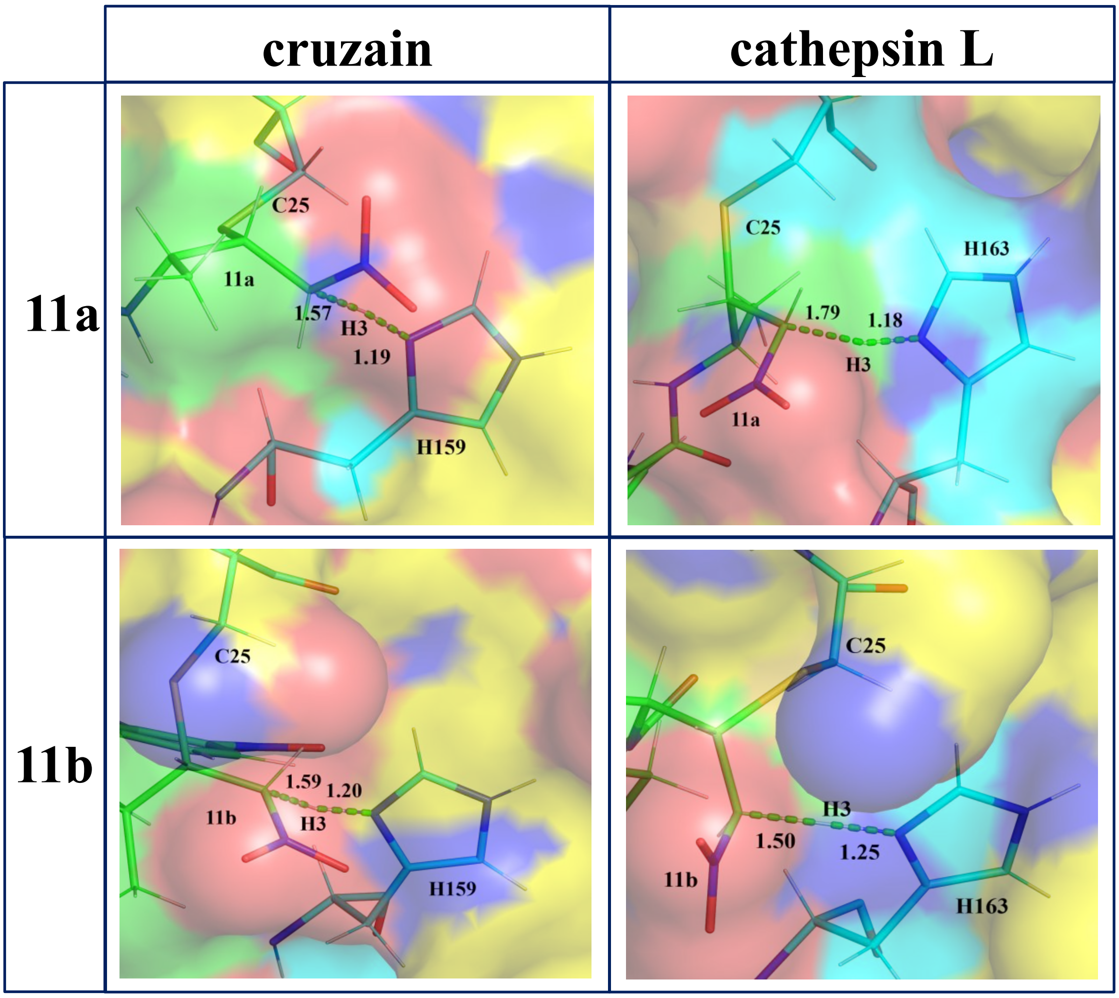

Figure S14. Detail of M06-2X/6-31+G(d,p)/MM optimized structures of TS2 located along the inhibition of cruzain (left panels) and cathepsin L (right panels) by **11a** (top panels) and **11b** (bottom panels). Key distances are in Å.

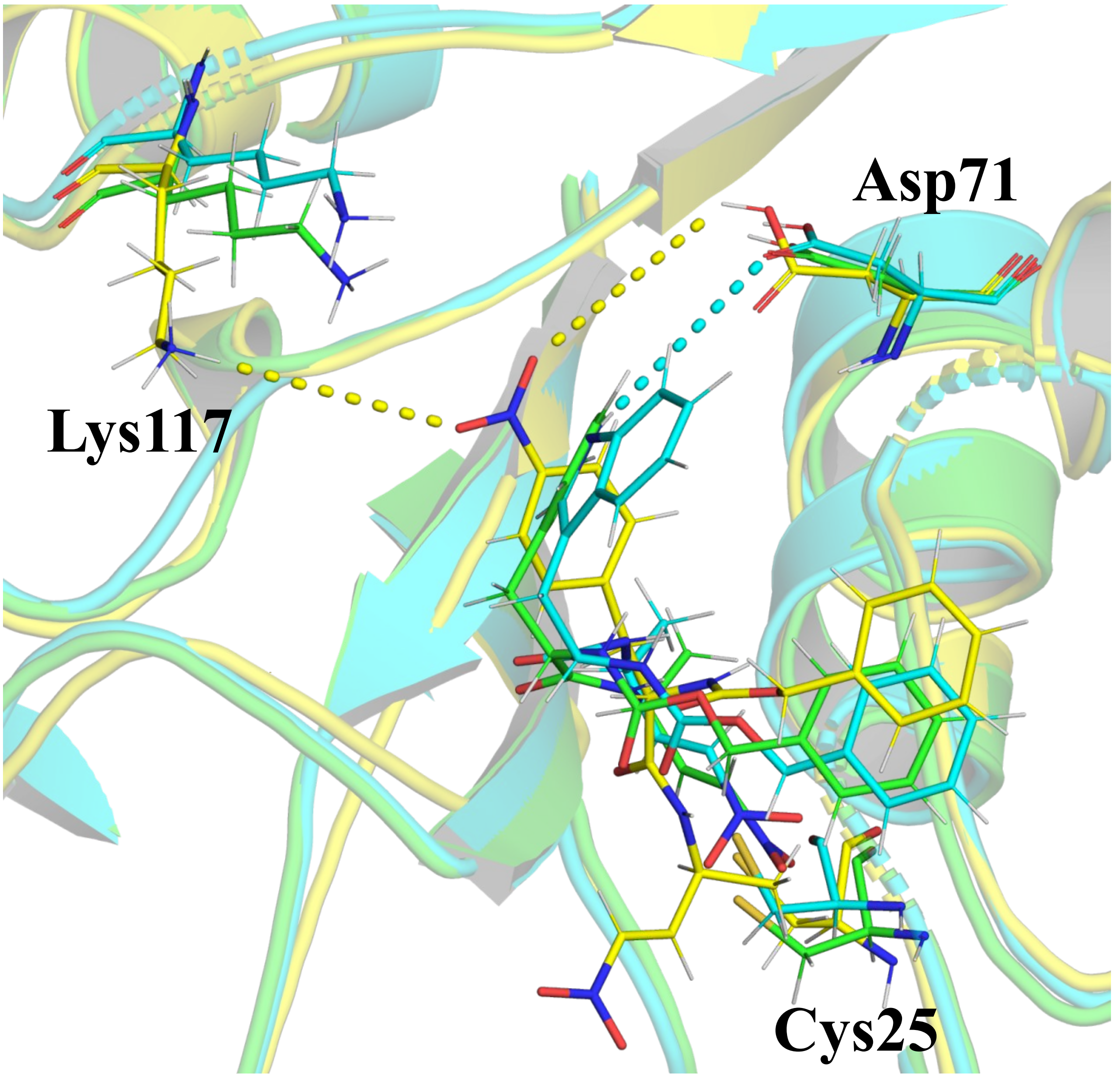

Figure S15. Overlay of the structures of **E·I** located along the inhibition of cathepsin L by **11** (in green), **11a** (in cyan) and **11b** (in yellow). Detail of the main hydrogen bond interactions between the P2 position of the inhibitor and S2 pocket of the cathepsin L.

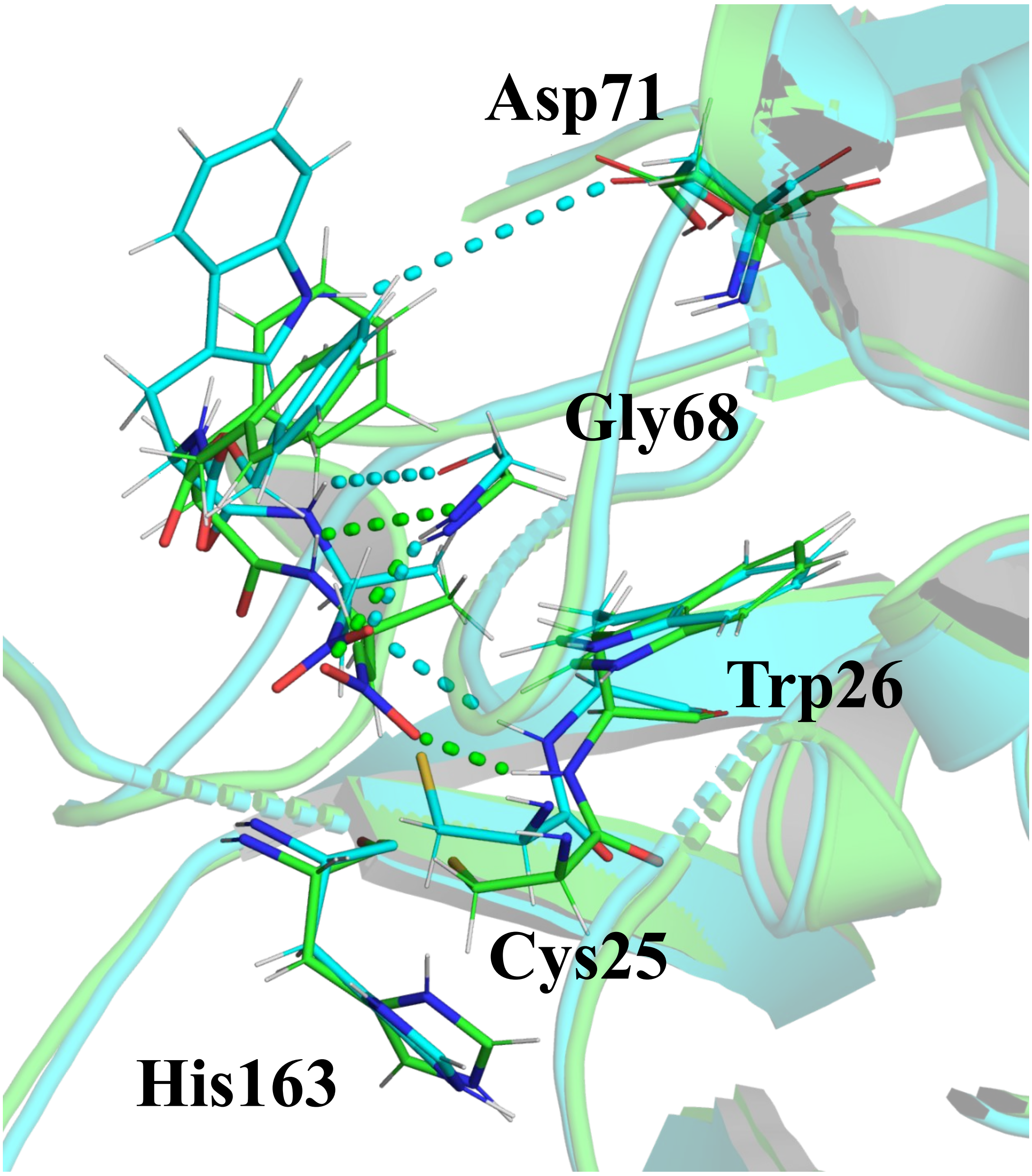

Figure S16. Overlay of the structures of **E·I** located along the inhibition of cathepsin L by **11** (in green) and **11a** (in cyan). Detail of the main hydrogen bond interactions between inhibitors and cathepsin L.

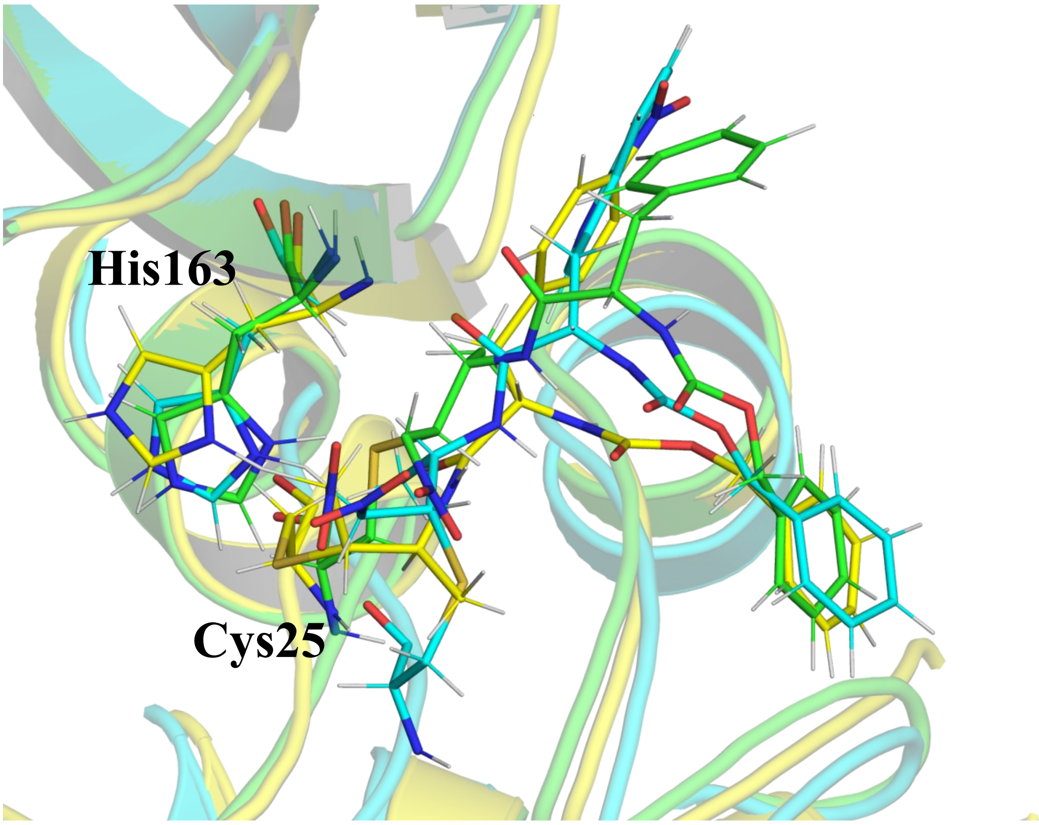

Figure S17. Overlay of the structures of TS2 located along the inhibition of cathepsin L by **11** (in green), **11a** (in cyan) and **11b** (in yellow).

III- Characterization data for synthesized compounds

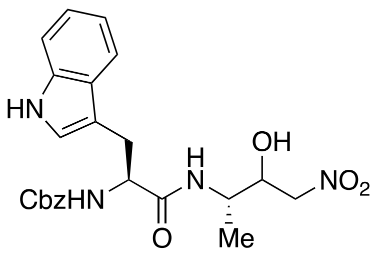

Benzyl ((2*S*)-1-(((2*S*)-3-hydroxy-4-nitrobutan-2-yl)amino)-3-(1*H*-indol-3-yl)-1-oxopropan-2 yl)carbamate.

White solid (m.p. 198-200 °C)

HRMS m/z calcd. for $C_{23}H_{26}N_4O_6Na$ [M + Na$^+$]: 477.1743, found: 477.1750.

^{1}H NMR (400 MHz, $CDCl_3$) δ 8.34 (s, 1H), 7.59 (dd, J = 13.5, 7.8 Hz, 1H), 7.40 – 7.27 (m, 6H), 7.18 (dd, J = 14.5, 7.3 Hz, 1H), 7.13 – 7.05 (m, 1H), 7.01 (d, J = 12.5 Hz, 1H), 6.28 – 5.92 (m, 1H), 5.65 (s, 1H), 5.07 (s, 2H), 4.52 – 4.37 (m, 1H), 4.24 – 4.07 (m, 1H), 4.07 – 3.91 (m, 1H), 3.91 – 3.70 (m, 1H), 3.70 – 3.36 (m, 1H), 3.32 – 3.19 (m, 1H), 3.19 – 3.08 (m, 1H), 1.92 (s, 1H), 1.02 (d, J = 5.4 Hz, 3H) ppm.

^{13}C NMR (101 MHz, $CDCl_3$) δ 171.78, 156.28, 136.37, 136.11, 128.71, 128.45, 128.24, 127.17, 123.45, 122.62, 120.04, 118.76, 111.72, 110.20, 77.36, 70.81, 67.41, 56.17, 47.19, 29.82, 17.35 ppm.

IR d 3395, 3299, 2967, 2922, 1700, 1654, 1551, 1454, 1342, 1230, 1126, 1081, 1044, 742, 697 cm^{-1}.

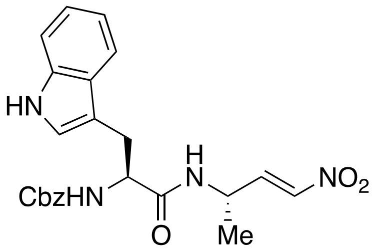

Benzyl ((*S*)-3-(1*H*-indol-3-yl)-1-(((*S*,*E*)-4-nitrobut-3-en-2-yl)amino)-1-oxopropan-2-yl)carbamate 11a.

Viscous yellow oil

α^{23}_D= +0.4 ($CHCl_3$, c = 4)

HRMS m/z calcd. for $C_{23}H_{25}N_4O_5$ [M + H$^+$]: 437.1825, found: 437.1830.

^{1}H NMR (300 MHz, $CDCl_3$) δ 8.28 (s, 1H), 7.59 (d, J = 7.9 Hz, 1H), 7.42 – 7.28 (m, 6H), 7.20 (t, J = 7.4 Hz, 1H), 7.11 (t, J = 7.2 Hz, 1H), 7.00 (dd, J = 6.8, 1.9 Hz, 1H), 6.96 – 6.33 (m, 2H), 5.86 (d, J = 34.9 Hz, 1H), 5.53 (d, J = 19.2 Hz, 1H), 5.09 (s, J = 3.1 Hz, 2H), 4.68 – 4.55 (m, 1H), 4.55 – 4.40 (m, 1H), 3.33 (ddd, J = 19.6, 14.3, 5.3 Hz, 1H), 3.23 – 3.04 (m, 1H), 0.98 (d, J = 7.1 Hz, 3H) ppm.

^{13}C NMR (101 MHz, $CDCl_3$) δ 171.13, 156.18, 141.95, 139.37, 136.43, 136.06, 128.72, 128.47, 128.23, 127.00, 123.43, 122.69, 120.07, 118.71, 111.84, 110.24, 67.42, 55.73, 43.24, 29.10, 19.18 ppm.

IR δ 3245, 2958, 2922, 1702, 1655, 1550, 1453, 1225, 1120, 1076, 1042, 740, 697 cm^{-1}.

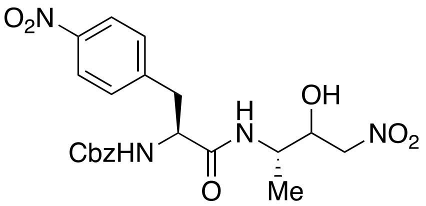

Benzyl ((2*S*)-1-(((2*S*)-3-hydroxy-4-nitrobutan-2-yl)amino)-3-(4-nitrophenyl)-1-oxopropan-2-yl)carbamate

White solid (m.p. 160-162 °C)

HRMS m/z calcd. for $C_{21}H_{26}N_4O_8$ [M + H$^+$]: 461.1669, found: 461.1672.

^1H NMR (400 MHz, $CDCl_3$) δ 8.12 (d, J = 7.6 Hz, 2H), 7.41 – 7.31 (m, 5H), 7.31 – 7.26 (m, 2H), 6.45 – 6.22 (m, 1H), 5.53 – 5.25 (m, 1H), 5.12 – 4.99 (m, 2H), 4.48 – 4.19 (m, 4H), 4.07 – 3.94 (m, 1H), 3.27 – 3.15 (m, 1H), 3.15 – 3.00 (m, 1H), 1.70 (s, 1H), 1.11 (d, J = 6.9 Hz, 3H) ppm.

^{13}C NMR (101 MHz, $CDCl_3$) δ 170.53, 156.19, 147.33, 144.08, 135.76, 130.33, 128.78, 128.69, 128.35, 124.01, 78.96, 71.30, 67.74, 47.83, 47.08, 37.96, 17.85 ppm.

IR d 3288, 2978, 1736, 1673, 1554, 1513, 1454, 1390, 1248, 1159, 1047, 951, 861, 779, 745 cm^{-1}.

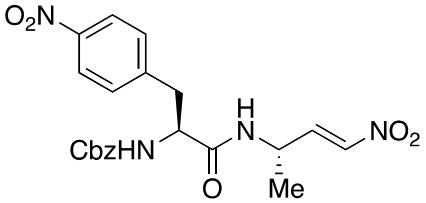

Benzyl ((*S*)-1-(((*S*,*E*)-4-nitrobut-3-en-2-yl)amino)-3-(4-nitrophenyl)-1-oxopropan-2-yl)carbamate 11b.

White solid (m. p. 192-193 °C)

HRMS m/z calcd. for $C_{21}H_{22}N_4O_7Na$ [M + Na$^+$]: 465.1392, found: 465.0386.

α^{23}_D= -1 ($CHCl_3$, c = 3.6)

^1H NMR (400 MHz, $CDCl_3$) δ 8.14 (dd, J = 8.7, 3.1 Hz, 2H), 7.43 – 7.28 (m, 7H), 7.12 – 6.73 (m, 2H), 6.23 (s, 1H), 5.29 – 5.19 (m, 1H), 5.12 – 5.02 (m, 2H), 4.78 – 4.64 (m, 1H), 4.46 – 4.35 (m, 1H), 3.32 – 3.20 (m, 1H), 3.17 – 3.09 (m, 1H), 1.29 (d, J = 7.1 Hz, 3H) ppm.

^{13}C NMR (101 MHz, d_6-DMSO) δ 170.22, 155.78, 146.27, 146.03, 144.14, 139.04, 136.91, 130.59, 128.20, 127.71, 127.51, 123.12, 65.32, 55.61, 42.91, 37.34, 19.23 ppm.

IR δ 3291, 2952, 2874, 1692, 1651, 1513, 1438, 1346, 1260, 1137, 1051, 954, 853, 731, 693 cm^{-1}.

IV- Copy of ^{1}H and ^{13}C-NMR Spectra

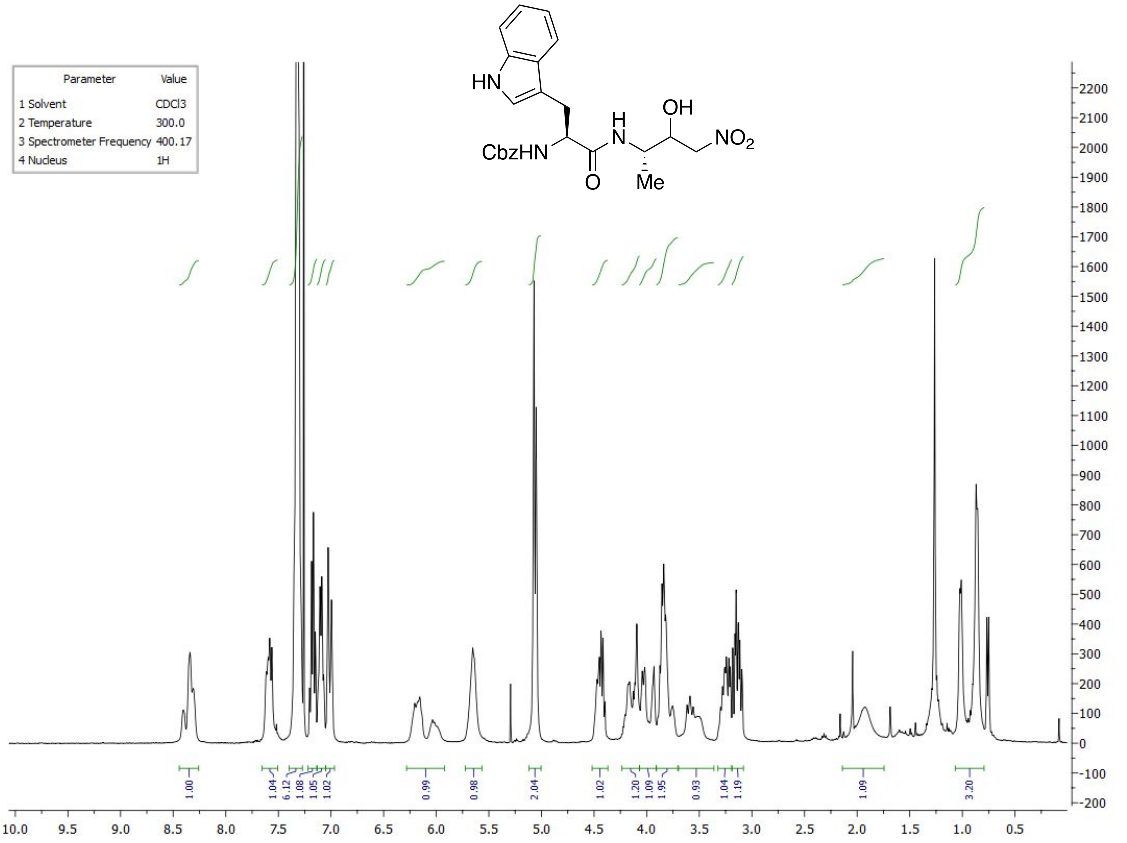

Figure S18. ^{1}H-NMR spectrum for nitroaldol precursor of inhibitor **11a.**

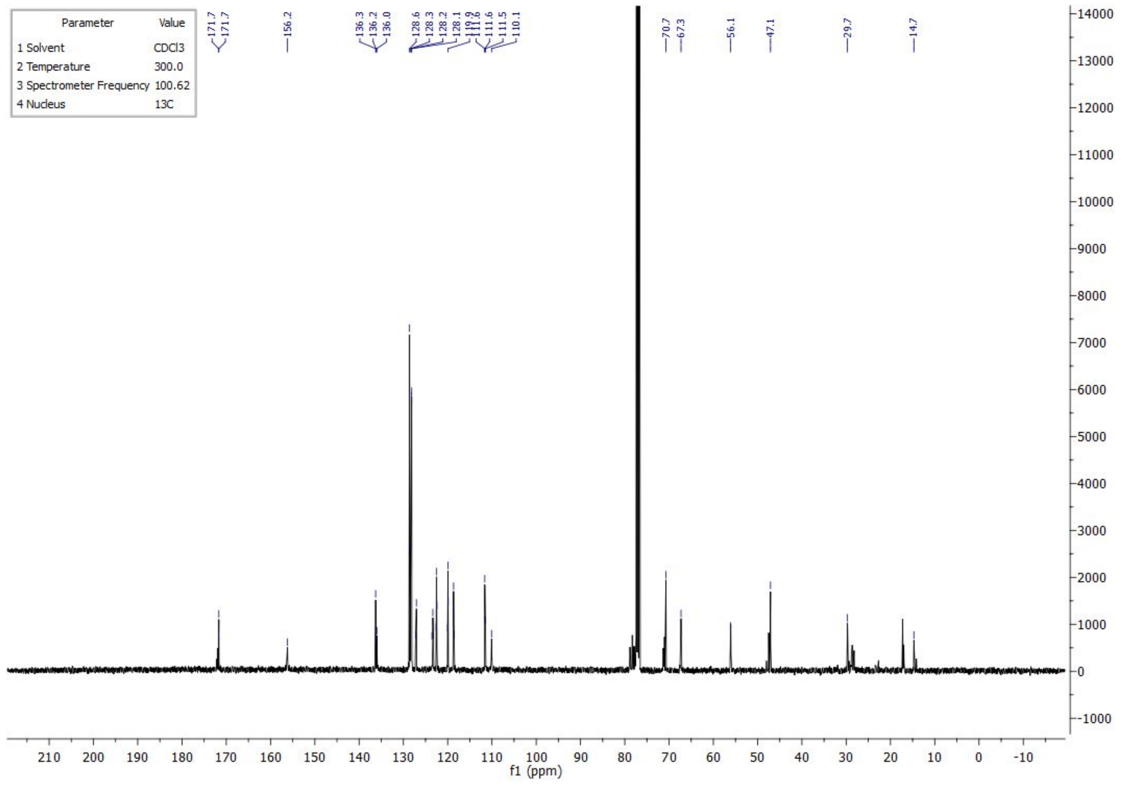

Figure S19. ^{13}C-NMR spectrum for nitroaldol precursor of inhibitor **11a.**

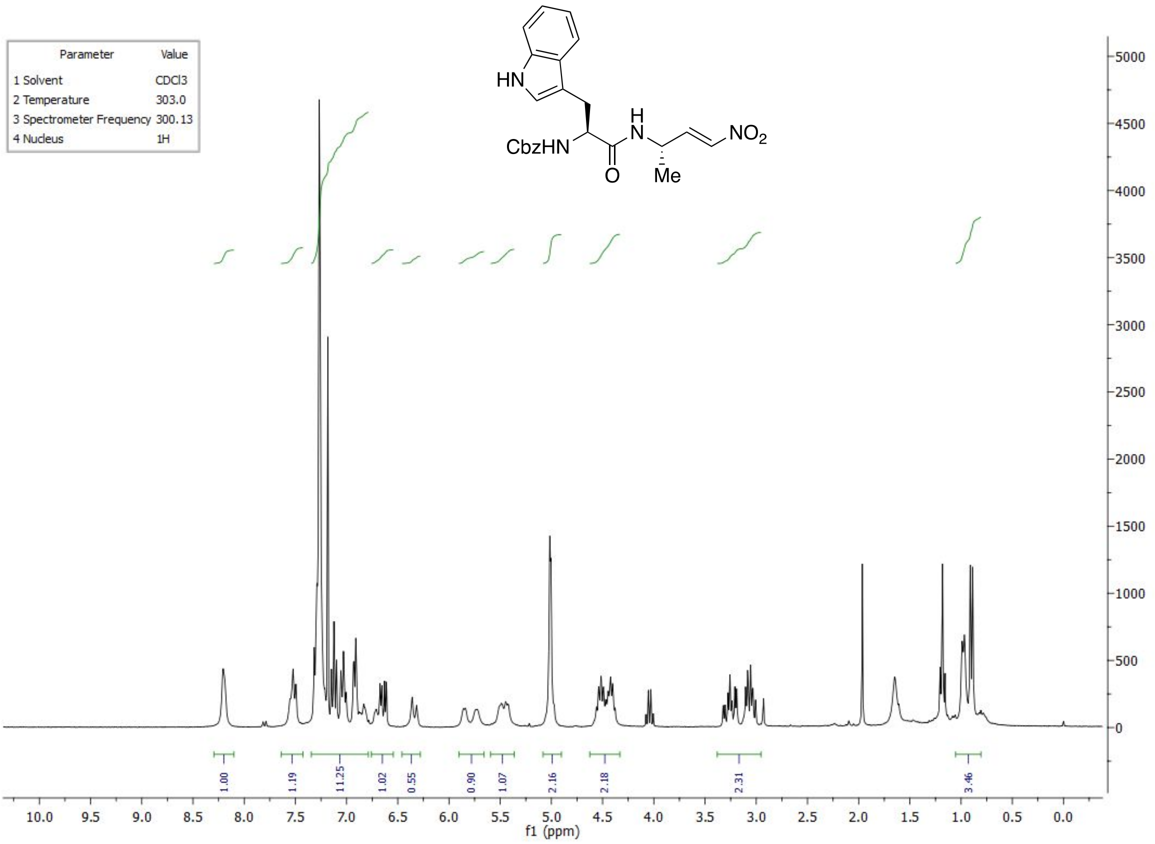

Figure S20. ^{1}H-NMR spectrum for inhibitor **11a.**

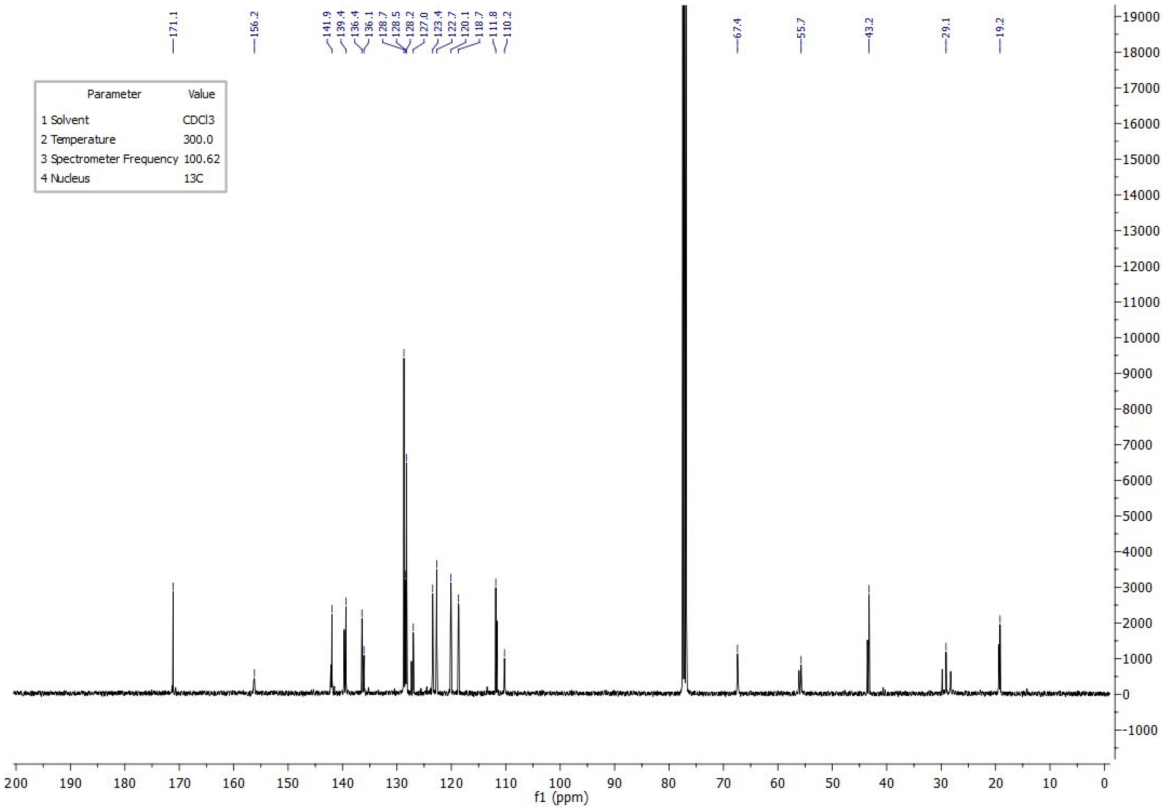

Figure S21. ^{13}C-NMR spectrum for inhibitor **11a.**

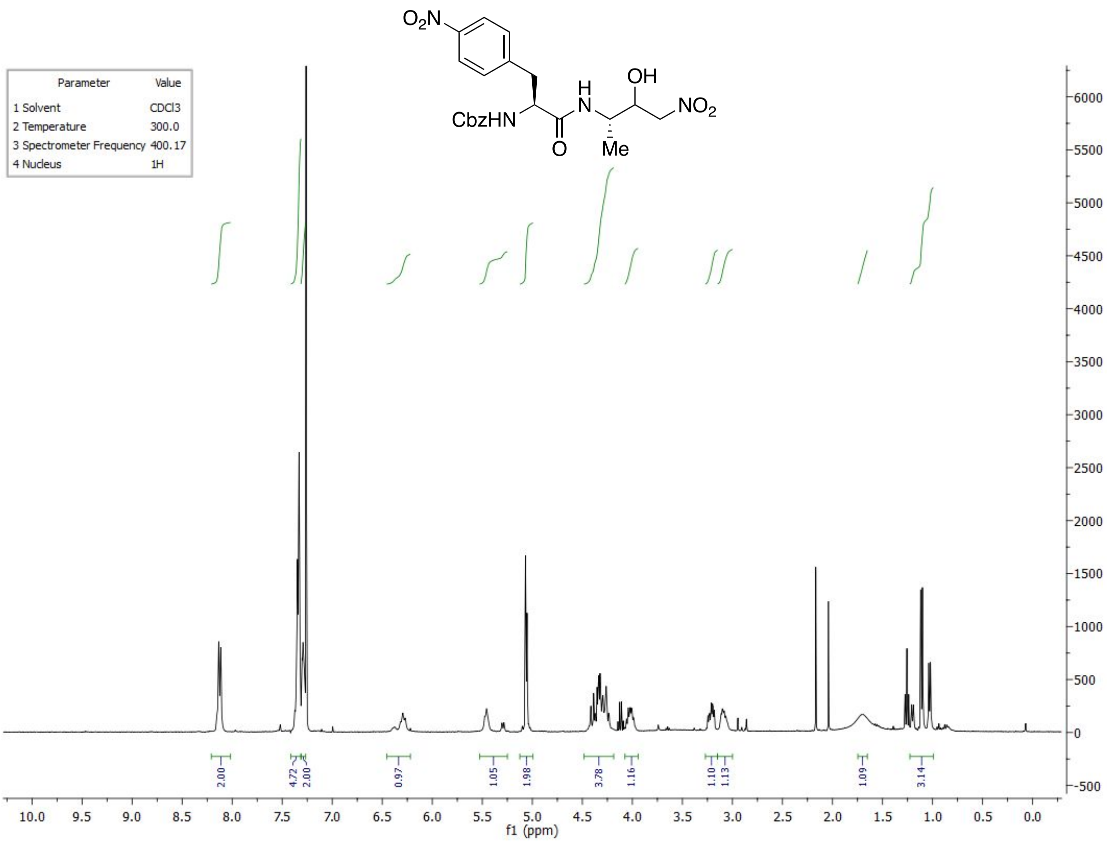

Figure S22. ^{1}H-NMR spectrum for nitroaldol precursor of inhibitor **11b.**

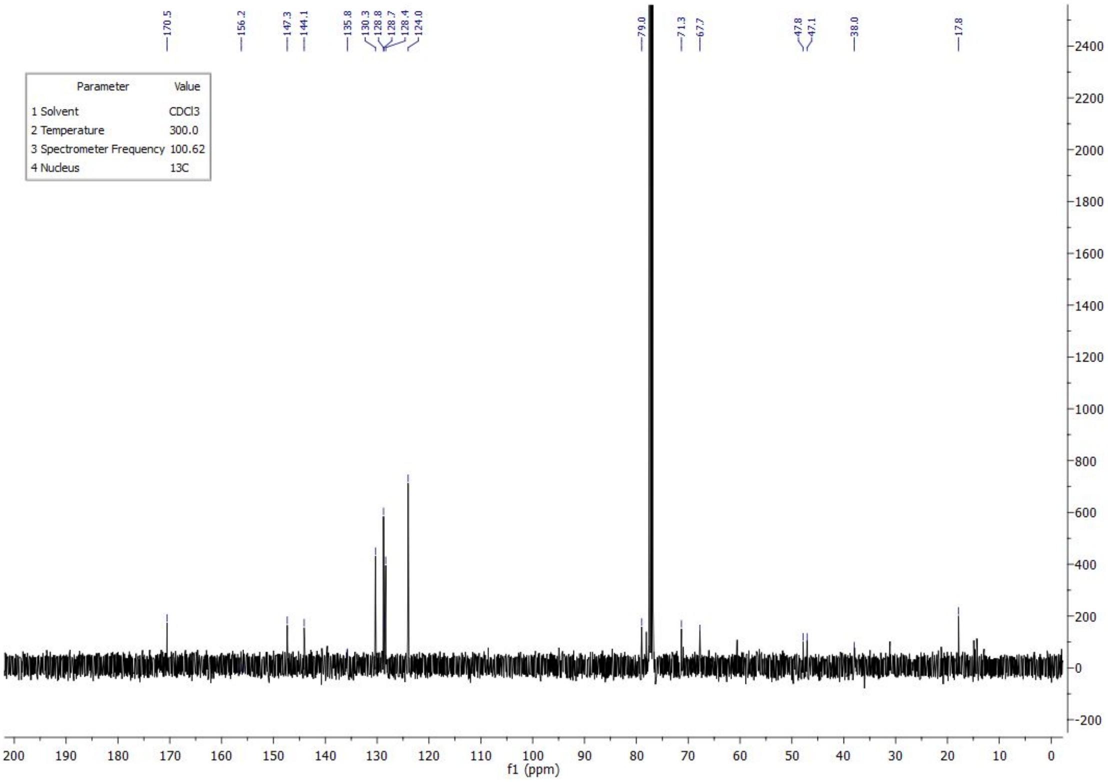

Figure S23. ^{13}C-NMR spectrum for nitroaldol precursor of inhibitor **11b.**

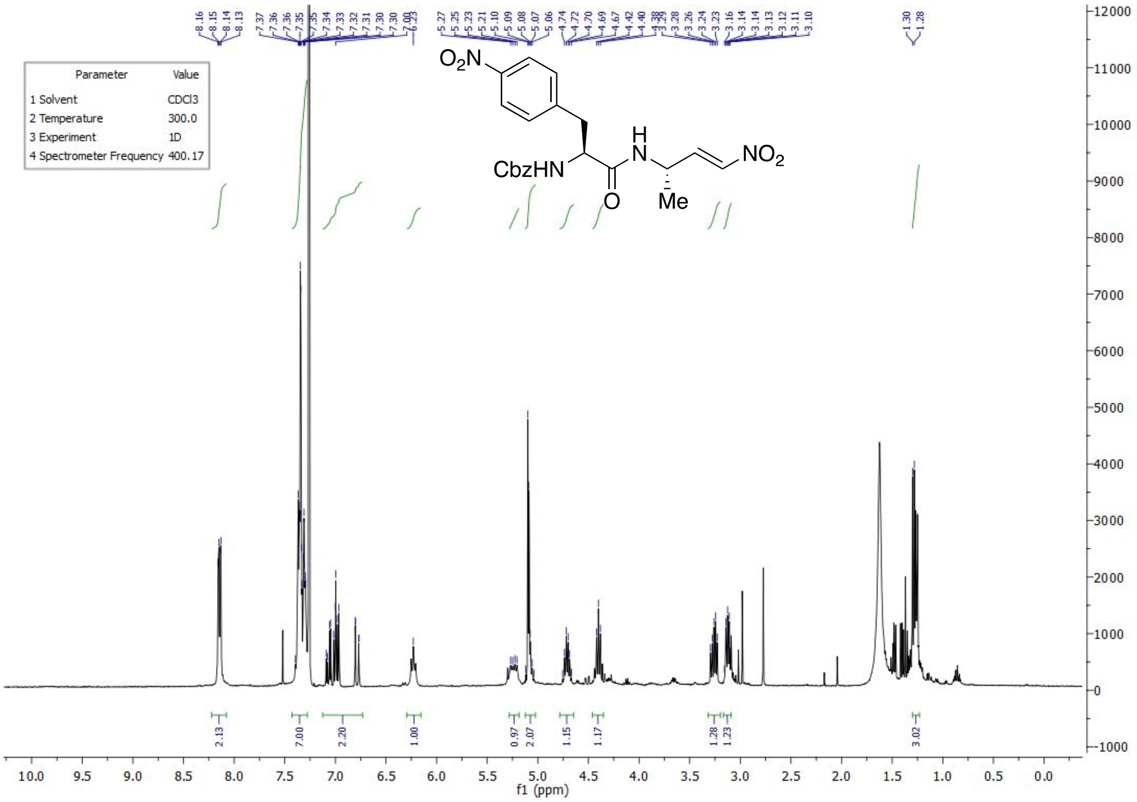

Figure S24. ^{1}H-NMR spectrum for nitroaldol precursor of inhibitor **11b.**

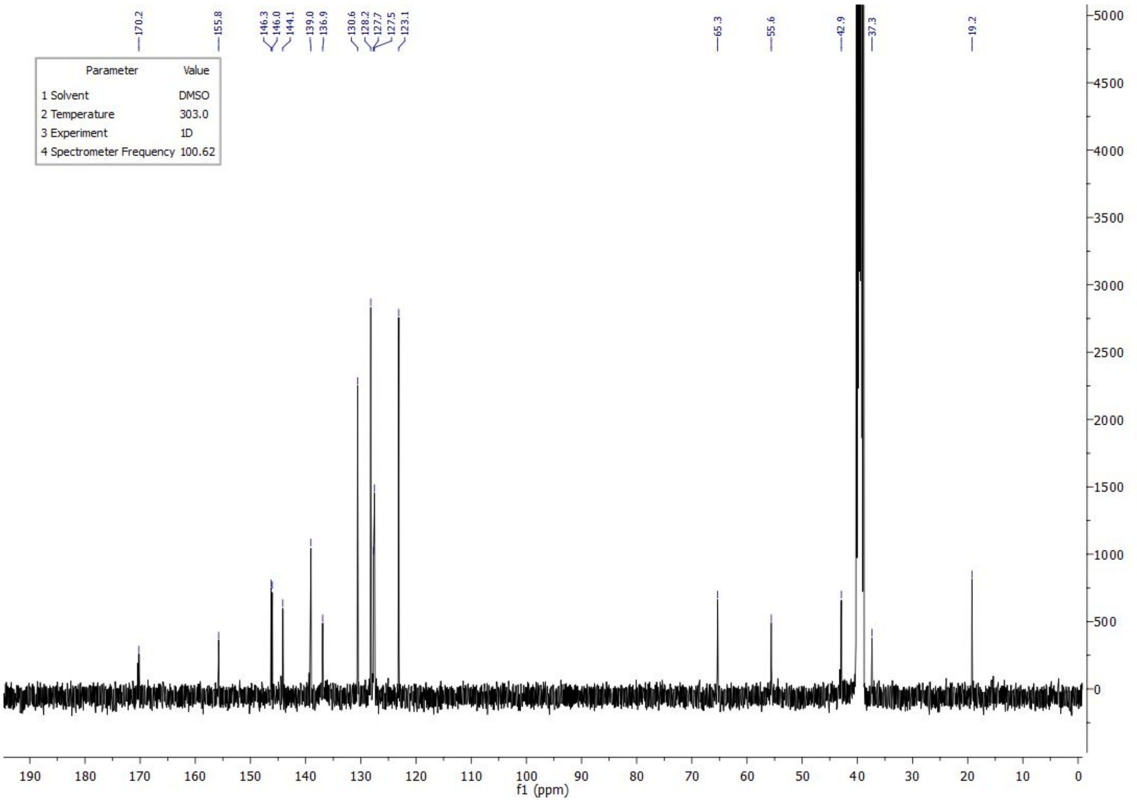

Figure S25. ^{13}C-NMR spectrum for nitroaldol precursor of inhibitor **11b.**

V- References

(1) Gillmor, S. A.; Craik, C. S.; Fletterick, R. J. Structural determinants of specificity in the cysteine protease cruzain. *Protein Sci.* **1997**, *6*, 1603-1611.

(2) Hardegger, L. A.; Kuhn, B.; Spinnler, B.; Anselm, L.; Ecabert, R.; Stihle, M.; Gsell, B.; Thoma, R.; Diez, J.; Benz, J.; Plancher, J.-M.; Hartmann, G.; Banner, D. W.; Haap, W.; Diederich, F. Systematic Investigation of Halogen Bonding in Protein–Ligand Interactions. *Angew. Chem. Int. Ed.* **2011**, *50*, 314-318.

(3) Arafet, K.; Ferrer, S.; Moliner, V. First Quantum Mechanics/Molecular Mechanics Studies of the Inhibition Mechanism of Cruzain by Peptidyl Halomethyl Ketones. *Biochemistry* **2015**, *54*, 3381-3391.

(4) Arafet, K.; Ferrer, S.; Gonzalez, F. V.; Moliner, V. Quantum mechanics/molecular mechanics studies of the mechanism of cysteine protease inhibition by peptidyl-2,3-epoxyketones. *Phys. Chem. Chem. Phys.* **2017**, *19*, 12740-12748.

(5) Arafet, K.; Ferrer, S.; Moliner, V. Computational Study of the Catalytic Mechanism of the Cruzain Cysteine Protease. *ACS Catalysis* **2017**, *7*, 1207-1215.

(6) Arafet, K.; Świderek, K.; Moliner, V. Computational Study of the Michaelis Complex Formation and the Effect on the Reaction Mechanism of Cruzain Cysteine Protease. *ACS Omega* **2018**, *3*, 18613-18622.

(7) Arafet, K.; González, F. V.; Moliner, V. Quantum Mechanics/Molecular Mechanics Studies of the Mechanism of Cysteine Proteases Inhibition by Dipeptidyl Nitroalkenes. *Chem. Eur. J.* **2020**, *26*, 2002-2012.

(8) Salomon-Ferrer, R.; Case, D. A.; Walker, R. C. An overview of the Amber biomolecular simulation package. *WIREs Computational Molecular Science* **2013**, *3*, 198-210.

(9) Olsson, M. H. M.; Sondergaard, C. R.; Rostkowski, M.; Jensen, J. H. PROPKA3: Consistent Treatment of Internal and Surface Residues in Empirical pKa Predictions. *J. Chem. Theory Comput.* **2011**, *7*, 525-537.

(10) Wang, J.; Wang, W.; Kollman, P. A.; Case, D. A. Automatic atom type and bond type perception in molecular mechanical calculations. *Journal of Molecular Graphics & Modelling* **2006**, *25*, 247-260.

(11) Jorgensen, W. L.; Chandrasekhar, J.; Madura, J. D.; Impey, R. W.; Klein, M. L. Comparison of Simple Potential Functions for Simulating Liquid Water. *J. Chem. Phys.* **1983**, *79*, 926-935.

(12) Duan, Y.; Wu, C.; Chowdhury, S.; Lee, M. C.; Xiong, G. M.; Zhang, W.; Yang, R.; Cieplak, P.; Luo, R.; Lee, T.; Caldwell, J.; Wang, J. M.; Kollman, P. A Point-charge Force Field for Molecular Mechanics Simulations of Proteins Based on Condensed-phase Quantum Mechanical Calculations. *J. Comput. Chem.* **2003**, *24*, 1999-2012.

(13) Phillips, J. C.; Braun, R.; Wang, W.; Gumbart, J.; Tajkhorshid, E.; Villa, E.; Chipot, C.; Skeel, R. D.; Kalé, L.; Schulten, K. Scalable Molecular Dynamics with NAMD. *J. Comput. Chem.* **2005**, *26*, 1781-1802.

(14) Grest, G. S.; Kremer, K. Molecular Dynamics Simulation for Polymers in the Presence of a Heat Bath. *Phys. Rev. A* **1986**, *33*, 3628-3631.

(15) Roe, D. R.; Cheatham, T. E. PTRAJ and CPPTRAJ: Software for Processing and Analysis of Molecular Dynamics Trajectory Data. *J. Chem. Theory Comput.* **2013**, *9*, 3084-3095.

(16) Nam, K.; Cui, Q.; Gao, J.; York, D. M. Specific reaction parametrization of the AM1/d Hamiltonian for phosphoryl transfer reactions: H, O, and P atoms. *J. Chem. Theory Comput.* **2007**, *3*, 486-504.
(17) Arafet, K.; Ferrer, S.; Martí, S.; Moliner, V. Quantum Mechanics/Molecular Mechanics Studies of the Mechanism of Falcipain-2 Inhibition by the Epoxysuccinate E64. *Biochemistry* **2014**, *53*, 3336-3346.
(18) Arafet, K.; González, F. V.; Moliner, V. Elucidating the Dual Mode of Action of Dipeptidyl Enoates in the Inhibition of Rhodesain Cysteine Proteases. *Chemistry – A European Journal* **2021**, *27*, 10142-10150.
(19) Jorgensen, W. L.; Maxwell, D. S.; TiradoRives, J. Development and testing of the OPLS all-atom force field on conformational energetics and properties of organic liquids. *J. Am. Chem. Soc.* **1996**, *118*, 11225-11236.
(20) Field, M. J.; Bash, P. A.; Karplus, M. A combined quantum-mechanical and molecular mechanical potential for molecular-dynamics simulations. *J. Comput. Chem.* **1990**, *11*, 700-733.
(21) Turner, A. J.; Moliner, V.; Williams, I. H. Transition-state Structural Refinement with GRACE and CHARMM: Flexible QM/MM Modelling for Lactate Dehydrogenase. *Phys. Chem. Chem. Phys.* **1999**, *1*, 1323-1331.
(22) Martí, S.; Moliner, V.; Tuñón, I. Improving the QM/MM Description of Chemical Processes: A Dual Level Strategy to Explore the Potential Energy Surface in Very Large Systems. *J. Chem. Theory Comput.* **2005**, *1*, 1008-1016.
(23) Kumar, S.; Bouzida, D.; Swendsen, R. H.; Kollman, P. A.; Rosenberg, J. M. The weighted histogram analysis method for free-energy calculations on biomolecules. 1. The method. *J. Comp. Chem.* **1992**, *13*, 1011-1021.
(24) Torrie, G. M.; Valleau, J. P. Non-physical sampling distributions in monte-carlo free-energy estimation - Umbrella Sampling. *J. Comp. Phys.* **1977**, *23*, 187-199.
(25) Field, M. J., *A Practical Introduction to the Simulation of Molecular Systems*, Second Edition ed., Cambrige University Press, Cambrige, UK, **2007**.
(26) Zhao, Y.; Truhlar, D. G. The M06 Suite of Density Functionals for Main Group Thermochemistry, Thermochemical Kinetics, Noncovalent Interactions, Excited States, and Transition Elements: Two New Functionals and Systematic Testing of Four M06-class Functionals and 12 Other Functionals. *Theor. Chem. Acc.* **2008**, *120*, 215-241.
(27) Hehre, W. J.; Radom, L.; Schleyer, P. V. R.; Pople, J. A., *Ab Initio Molecular Orbital Theory*, John Wiley, New York, **1986**.
(28) Lynch, B. J., Zhao, Y., Truhlar, D. G. Effectiveness of Diffuse Basis Functions for Calculating Relative Energies by Density Functional Theory. *J. Phys. Chem. A* **2003**, *107*, 1384-1388.
(29) M. J. Frisch; G. W. Trucks; H. B. Schlegel; G. E. Scuseria; M. A. Robb; J. R. Cheeseman; G. Scalmani; V. Barone; B. Mennucci; G. A. Petersson; H. Nakatsuji; M. Caricato, X. L., H. P. Hratchian, A. F. Izmaylov, J. Bloino, G. Zheng, J. L. Sonnenberg, M. Hada, M. Ehara, K. Toyota, R. Fukuda, J. Hasegawa, M. Ishida, T. Nakajima, Y. Honda, O. Kitao, H. Nakai, T. Vreven, J. A. Montgomery, Jr., J. E. Peralta, F. Ogliaro, M. Bearpark, J. J. Heyd, E. Brothers, K. N. Kudin, V. N. Staroverov, R. Kobayashi, J. Normand, K. Raghavachari, A. Rendell, J. C. Burant, S. S. Iyengar, J. Tomasi, M. Cossi, N. Rega, J. M. Millam, M. Klene, J. E. Knox, J. B. Cross, V. Bakken, C. Adamo, J. Jaramillo, R. Gomperts, R. E. Stratmann, O. Yazyev, A. J. Austin, R. Cammi, C. Pomelli, J. W. Ochterski, R. L. Martin, K. Morokuma, V. G. Zakrzewski, G. A. Voth, P. Salvador, J. J. Dannenberg, S.

Dapprich, A. D. Daniels, Ö. Farkas, J. B. Foresman, J. V. Ortiz, J. Cioslowski, D. J. Fox Gaussian 09 (Revision A.1), **2009**.

(30) Field, M. J.; Albe, M.; Bret, C.; Proust-De Martin, F.; Thomas, A. The Dynamo library for molecular simulations using hybrid quantum mechanical and molecular mechanical potentials. *J. Comp. Chem.* **2000**, *21*, 1088-1100.
